# Structural Reshaping of the Zinc-Finger Domain of the SARS-CoV-2 nsp13 Protein Using Bismuth(III) Ions: A Multilevel Computational Study

**DOI:** 10.1021/acs.inorgchem.2c02685

**Published:** 2022-09-20

**Authors:** Iogann Tolbatov, Loriano Storchi, Alessandro Marrone

**Affiliations:** † Institut de Chimie Moleculaire de L’Université de Bourgogne (ICMUB), Université de Bourgogne Franche-Comté (UBFC), Avenue Alain Savary 9, Dijon 21000, France; ‡ Dipartimento di Farmacia, Università“G D’Annunzio” di Chieti-Pescara, Via Dei Vestini 31, Chieti 66100, Italy

## Abstract

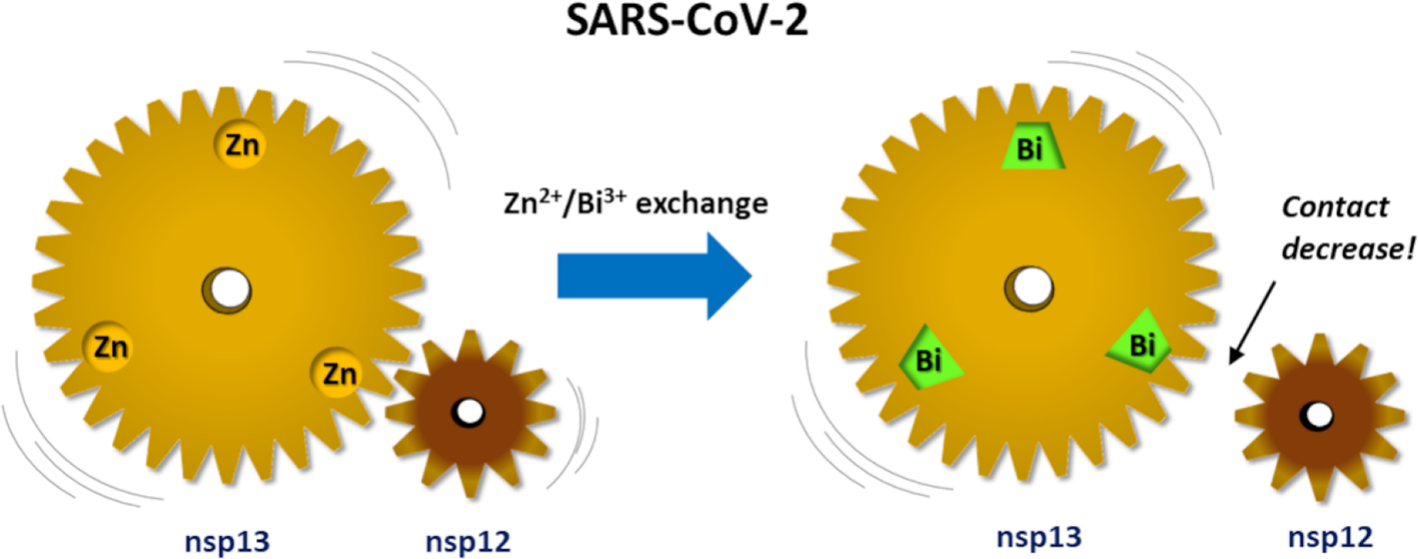

The identification of novel therapeutics against the pandemic SARS-CoV-2 infection is an indispensable new address of current scientific research. In the search for anti-SARS-CoV-2 agents as alternatives to the vaccine or immune therapeutics whose efficacy naturally degrades with the occurrence of new variants, the salts of Bi^3+^ have been found to decrease the activity of the Zn^2+^-dependent non-structural protein 13 (nsp13) helicase, a key component of the SARS-CoV-2 molecular tool kit. Here, we present a multilevel computational investigation based on the articulation of DFT calculations, classical MD simulations, and MIF analyses, focused on the examination of the effects of Bi^3+^/Zn^2+^ exchange on the structure and molecular interaction features of the nsp13 protein. Our calculations confirmed that Bi^3+^ ions can replace Zn^2+^ in the zinc-finger metal centers and cause slight but appreciable structural modifications in the zinc-binding domain of nsp13. Nevertheless, by employing an in-house-developed ATOMIF tool, we evidenced that such a Bi^3+^/Zn^2+^ exchange may decrease the extension of a specific hydrophobic portion of nsp13, responsible for the interaction with the nsp12 protein. The present study provides for a detailed, atomistic insight into the potential anti-SARS-CoV-2 activity of Bi^3+^ and, more generally, evidences the hampering of the nsp13–nsp12 interaction as a plausible therapeutic strategy.

## Abstract 2

The Zn^2+^/Bi^3+^ exchange in the zinc finger domains of SARS-CoV-2 nsp13 hampers the viral machinery

## Introduction

As sadly ascertained worldwide, coronavirus disease 2019 (COVID-19) has resulted in several million deaths by the end of 2021.^1^ The sheer scale of this unprecedented epidemics has given an incentive of an unparalleled search for effective drugs against its cause, the severe acute respiratory syndrome coronavirus 2 (SARS-CoV-2). A plethora of possible targets were identified in SARS-CoV-2,^2−4^ including RNA-dependent RNA polymerase, 3-chymotrypsin-like protease, and papain-like protease and other proteins crucial for viral entry, replication, and pathogenesis, including structural and nonstructural proteins.^5^ Many studies suggest that the most promising mechanisms in the virus to be attacked are the viral entry and replication.^6−9^ Moreover, computational studies utilizing molecular modeling and virtual screening against known antivirals have been employed for the determination of candidate SARS-CoV-2 helicase inhibitors.^10,11^

Non-structural protein 13 (nsp13), a 67 kDa protein, pertains to the helicase superfamily 1B and catalyzes the unwinding of double-stranded DNA or RNA by means of the hydrolysis energy of nucleotide triphosphate.^12^ This protein operates both on RNA and DNA, exhibiting relatively weak non-processive helicase activity in comparison with other enzymes from the superfamily 1B.^13,14^ A joint activity has been observed in nsp13 and the viral RNA-dependent RNA polymerase nsp12,^15^ functioning in conjunction with the complex nsp7/nsp8/nsp12 for replication transcription.^16^ Furthermore, nsp13 plays a crucial role also in the viral 5′ mRNA cap formation by performing the RNA 5′ triphosphatase activity.^17^

Several computational studies focused on nsp13. The importance of targeting nsp13 was underlined by studies which combined homology modeling and molecular dynamics simulations.^18,19^ The virtual screening of chemical compounds against nsp13 has identified potential inhibitors.^19−21^ The study focused on mapping major SARS-CoV-2 drug targets and assessment of druggability, using computational fragment screening, and identified favorable allosteric sites in the zinc-binding domains (ZBDs) that were proposed for the virtual or biophysical fragment screening.^22,23^ Indeed, the structural analysis^15^ showed that SARS coronavirus helicase contains three canonical zinc fingers (ZFs), including ZF 1 (Cys5, Cys8, Cys26, and Cys29), ZF 2 (Cys16, Cys19, His33, and His39), and ZF 3 (Cys50, Cys55, Cys72, and His75). These ZFs were targeted using bismuth salts in two recent studies^24,25^ and another study dating 15 years back.^26^ It was shown that bismuth salts, including ranitidine bismuth citrate (RBC) and bismuth potassium citrate (BPC), inhibit helicases SCV and nsp13 in coronaviruses SARS and SARS-CoV-2, respectively. The antiviral properties of RBC were expressly focused on in an investigation, showing that this compound strongly blocks both in vivo and in vitro replication of SARS-CoV-2 in human and animal cell lines and in an established golden Syrian hamster model by hampering the normal activity of helicase nsp13.^25^ This bismuth-based drug exhibited minor cytotoxicity and safeguarded the cells infected with SARS-CoV-2 with a high selectivity index of 975. RBC inhibited both the ATPase (IC50 = 0.69 μM) and DNA unwinding (IC50 = 0.70 μM) functions of nsp13 by means of an irreversible substitution of Zn^2+^ ions in this enzyme with Bi^3+^ ions, according to in vitro studies. This resulted into a complete suppression of the SARS-CoV-2 replication and diminished viral loads in both upper and lower respiratory tracts in a golden Syrian hamster model.

As shown in a study by Yuan S. et al.,^25^ the usage of RBC results in the substitution of Zn^2+^ cations with Bi^3+^ cations and the release of the zinc ions. This is a very important experimental observation which suggests that RBC may serve as a broad-spectrum inhibitor against coronavirus since the ZF motif is a key motif in coronavirus enzymes, which is highly conserved.

The chemical nature of the nsp13 impairment caused by the Zn^2+^ replacement with Bi^3+^ ions is probably multifaceted. The overall +3 increase in the positive charge may induce a different distribution of the ionized side chains within the structure of nsp13 ZBD, which in turn may either interfere with or fade the functionality of this domain. Indeed, several cryoelectron microscopy data by Darst et al.^27,28^ and by Lou et al.,^29^ deposited in the pdb archive, display the ZBD of nsp13 involved in the interaction with nsp12, which is a part of the replication and transcription complex and thus crucially implicated in the molecular mechanism of the SARS-CoV-2 cellular infection. Therefore, Zn^2+^ and Bi^3+^ are characterized by rather different coordination features: Zn^2+^ coordination with cysteine or histidine side chains is prevalently tetrahedral, whereas Bi^3 +^ coordination is expectedly affected by the presence of the 6s lone pair that leads to distortion.

We repute that the replacement of Zn^2+^ ions from the three metal binding sites of the nsp13 ZBD may potentially induce an internal strain, causing the different spatial arrangement of the coordinating residues around the Bi^3+^ ions.

Moreover, at physiological pH (∼7.4), the fraction of deprotonated Cys equals 5% (calculated for the side chain pKa value of 8.3); thus, the availability of both neutral and deprotonated cysteines in the zinc-binding motifs shows the possibility of the presence of multiple protonation states in which each cysteine can be either neutral or deprotonated. Thus, we hypothesize that the Zn^2+^/Bi^3+^ exchange may also affect the pKa of the coordinating cysteines and, in turn, remodel the structure of the involved metal binding sites.

In this study, a multilevel computational approach^30,31^ was employed to shed light on the structural alterations accompanying the process of Zn^2+^ substitution with Bi^3+^ in the nsp13 ZBD. DFT calculations were used for studying the thermodynamics of the Zn^2+^/Bi^3+^ exchange and for assessing the protonation state of the coordinating cysteines in either the Zn-bound or Bibound ZBD. Moreover, molecular dynamics simulations of the ZBD of nsp13 were performed by using the cationic dummy atom (CDA) approach to describe the coordination of either Zn^2+^ or Bi^3+^ metal centers. The MD trajectories of Zn-bound and Bi-bound ZBDs allowed us to assess the structural modifications induced by the metal exchange through the employment of the newly designed ATOMIF tool.^32,33^ The analyses of the electrostatic and hydrophobic molecular interaction fields evidenced that the Zn^2+^/Bi^3+^ exchange induces a reshaping of the nsp13 ZBD, which, in particular, affects the hydrophobic cleft involved in the binding with nsp12.

The computational outcomes presented here provide for a rationale to the nsp13 impairment caused by the Bi^3+^ treatment and evidence how changes in the metal coordination geometry may generate non-local structural impacts.

## Computational Details

### QM Calculations

All calculations were performed with the Gaussian 09 A.02 quantum chemistry package.^34^

Geometrical optimizations were carried out in solution by using ωB97X^35^ in combination with the def2SVP basis set.^36^ Frequency calculations were performed to verify the correct nature of the stationary points and to estimate zero-point energy (ZPE) and thermal corrections to thermodynamic properties. Single-point electronic energy calculations on the resulting geometries were then performed by using the range-corrected functional ωB97X with the basis set def2TZVP.^36^

DFT gives a good description of geometries and reaction profiles for complexes formed by either transition metals^37,38^ or heavy elements of the IV and V groups.^39−43^ Therefore, the ωB97X functional is known to reach a high accuracy in the calculation of electronic energies.^41,44,45^ The C-PCM continuum solvent method was used to describe the solvation.^46^

### Combined MD-MIF Study

**Figure 1:**
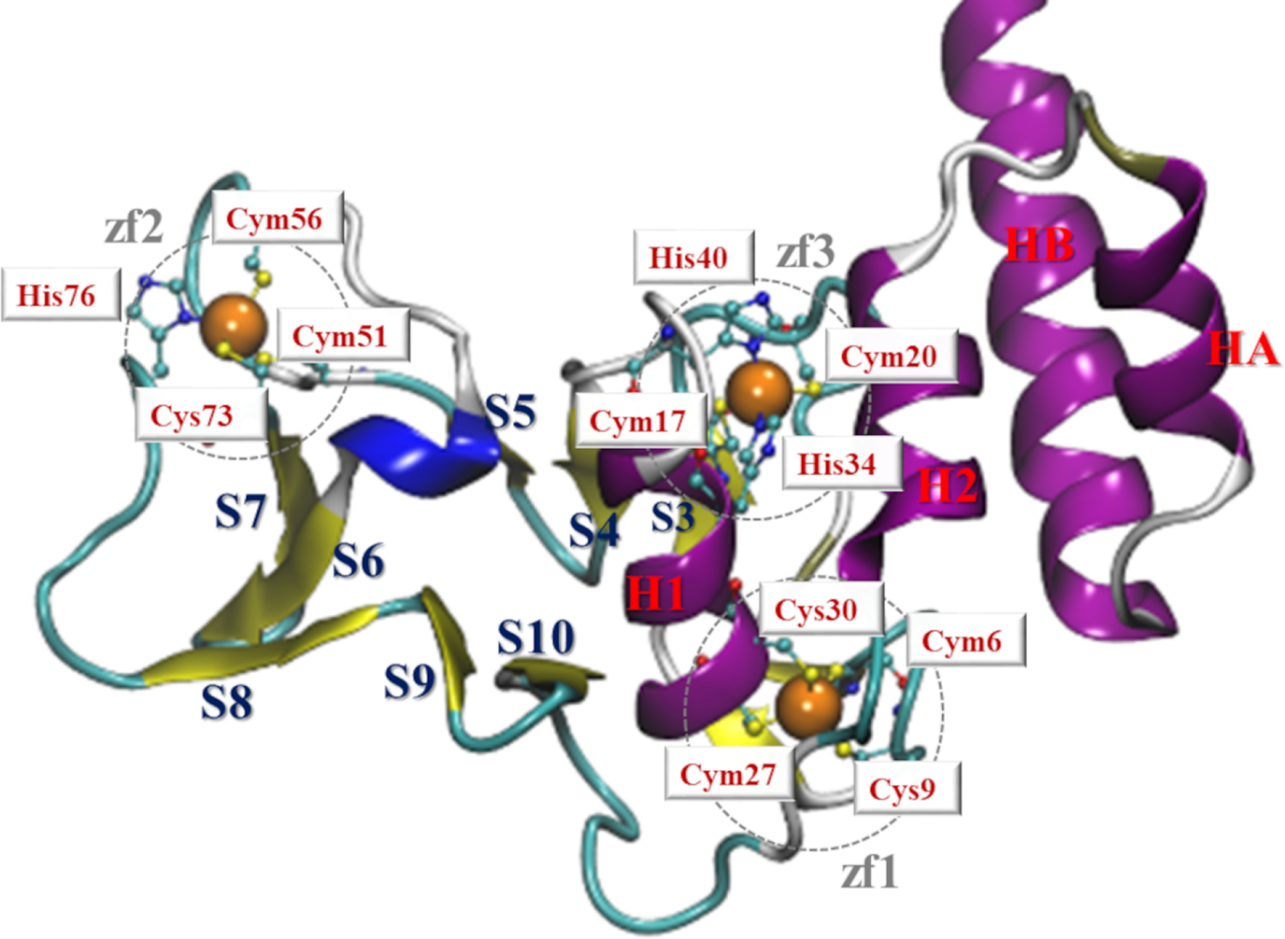
Rendition of the nsp13 ZBD. The three ZF sites, ZF1, ZF2, and ZF3, are evidenced (dashed circles). Zn^2+^ ions (orange sphere) and their coordinating residues (ball-and-sticks) are labeled (neutral or deprotonated cysteines are indicated with Cys or Cym, respectively). The α helix and β-sheet domains are also labeled (red and blue, respectively).

This investigation was performed using the ATOMIF tool^47^ for which a two-stage articulation is required. In the first stage, molecular dynamics is used to provide for a multiconfigurational representation of the studied molecular system. Hence, the starting structure of the ZF domain (ZFD) of the nsp13 protein (6zsl.pdb), recorded at 1.94 angstrom resolution, was simulated in the AMBER03 force field^48^ by using the Gromacs package.^49^ The ZFD was identified in the 2–150 segment of the nsp13 protein; such an excision included also two helix domains 117–124 (HA) and 128–150 (HB), placed immediately after the ZFD (2–111) in the primary sequence (Figure 1), which forms several intramolecular interactions with the ZFD. The three Zn^2+^ cations featuring the ZFD were assigned a cationic dummy atom (CDA) topology in which each metal center is bound to four dummy points placed in a tetrahedral geometry. The charge and mass of Zn were distributed within the metal center and the four dummy points (Du) as follows
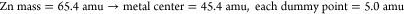

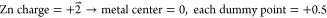


The CDA geometry around the metal center was ensured by bond and angle harmonic potentials (see Supporting Information), whose parameterization was obtained by adapting the CDA scheme of Pb^2+^ ions previously developed.^40^ More specifically, the same Pb-Du harmonic potential (reference distance = 0.7 Å and harmonic constant = 267520.0 kJ/mol/Å) was applied to all Zn-Du bonds, whereas the Du-Zn-Du angles were described using harmonic potentials with reference angle = 109.47° and a harmonic constant of 22670.0 kJ/mol/degree, in order to correctly render the tetrahedral geometry of the Zn^2+^ coordination. The most important difference in the CDA topology of Zn^2+^ compared to that previously reported^40^ is the inclusion of S-Du and N-Du harmonic potentials with reference distances of 1.68 and 1.31 Å, respectively, and harmonic constant = 1000 kJ/mol/Å to appropriately depict the partially covalent character of coordinative bonds formed by Cys or His residues. The inclusion of these S–Du and N–Du harmonic potentials also required the application of different sets of 12–6 Lennard–Jones parameters to Zn and Du atoms, compared to those previously reported for Pb and Du;^40^ in particular, σ values of 0.195998 and 0.100000 nm were assigned to Zn and Du, respectively, and ε values of 0.05230 and 0.01045 kJ/mol, in the same order. These LJ parameters were optimized by performing gas-phase optimization of reduced models of the zinc-ZFDs to provide minimal deviations from the corresponding DFT geometries (vide supra).

**Scheme 1:**
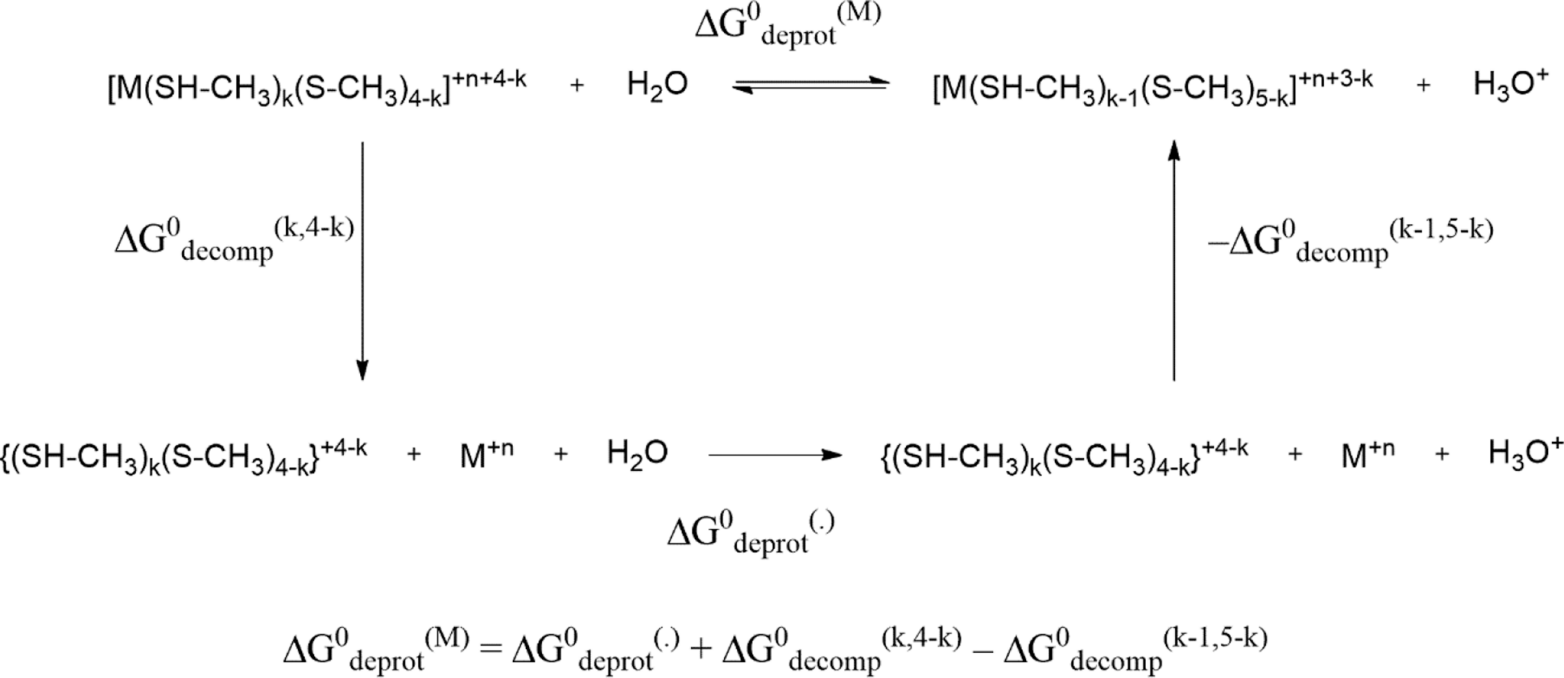
Thermodynamic Cycle Presenting the Deprotonation Free Energy of the Metal-Bound ZF1 Structure With *k* Neutral Cysteines in Terms of the Deprotonation Free Energy of the Unbound ZF1 and Decomplexation Free Energies.

The protein system was placed in a rectangular box with dimensions 76 × 76 × 76 Å^3^ and solvated with about 12600 water molecules (three-point water model TIP3P) at the typical density of liquid water at 300 K and 1.0 atm. Electrical neutrality was afterward achieved by replacing one water molecule with a Cl^–^ counterion. The simulation was performed adopting the following computational scheme: (1) local energy minimization, (2) an equilibration NVT run of 125 ps at 300K, and (3) a production run of 50 ns at 300 K in an isothermal/isobaric ensemble, using the velocity rescaling scheme for temperature and the isotropic Berendsen coupling scheme for pressure.^50^ The integration step was set to 0.5 fs. The LINCS algorithm was adopted to constrain all bond lengths,^51^ and the long-range electrostatics were computed by the particle mesh Ewald method.^52^ Trajectory analyses were carried out by using suitable Gromacs utilities with the support of either VMD^53^ or Maestro graphical interfaces.^54^ An ensemble of 800 protein conformations was extracted from the last 40 ns of trajectory corresponding to the stabilized system. The clustering method-labeled Gromos^55^ was then employed to sample a subset of representative configurations of the system.

An almost identical MD setting was employed in the simulation of the ZFD of nsp13, where all Zn^2+^ ions were replaced with Bi^3+^ ions. The only significant differences were represented by the different CDA topology used to describe the Bi^3+^-ion coordination (Figure 3). Indeed, based on the presence of the 6s electron pair, the coordination geometry of Bi^3+^ was assumed to be hemidirected, with Du-Bi-Du angles of either 82.6 or 214.8° (Figure 3). Therefore, the charge and mass of Bi were distributed within the metal center and four dummy points as it follows:
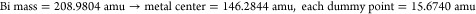

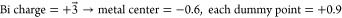


Analogous to the criteria adopted in the CDA scheme for the Zn^2+^ ion, we computed 30% of Bi mass to be distributed on the four dummy points, whereas the assignment of a negative charge on the Bi^3+^ center was carried out in order to consider the presence of the 6s^2^ electron pair.

In the second stage of ATOMIF analysis, the sets of representative conformations of both Zn^2+^- and Bi^3+^-bound ZBDs were processed using GRID software,^56^ where the interaction field is computed as the sum of the interactions of the uncharged hydrophobic probe (DRY) with all atoms of the target being immersed in the 3D grid, that is, the probe is moved point after point in the 3D grid. Finally, the computed DRY maps of the representative structures of both Zn- and Bi-bound ZBDs were analyzed to count high-field grid points, the average, and the total MIF per slice–summation of the MIF on all slice points divided or not, respectively, by the number of points–by allowing us to sketch the DRY fields with MIF profiles along the three Cartesian axes (*x*, *y*, *z*); details about the whole procedure can be found in refs (32) and (33) The ATOMIF analysis has been performed using a grid spacing of 1 Å.

Similar to the MIF, the MEP is defined as the interaction energy between a positively charged probe (+1) located at a given grid point and the atomic charges of the protein. Diversely from the MIF, a MEP provides information on the presence of positively or negatively charged regions. In this work, the MEP evaluation has been obtained using two different model equations: the Poisson–Boltzmann solver,^57,58^ including therefore the solvent effect, and the classical Coulomb potential in the gas phase (unitary dielectric constant). Finally, the computed MEP were used to perform pairwise comparisons via the Carbò index of the two sets of representative conformations.^33,59−61^ This procedure allows us to easily spot regions of similarity and dissimilarity between the two representative sets.

All protein–protein superimposition analyses were carried out by using the TM-align web server. The most representative conformations of Zn-bound and Bi-bound ZBD models (vide infra) were superimposed with the protein assembly retrieved from the pdb entry 7CXN,^29^ and the corresponding superimposed structures were graphically analyzed in the VMD workspace.^53^

## Results and Discussion

### ZFs

The available X-ray structures of the protein nsp13 ZBD display the presence of three Zn^2+^ binding sites (ZFs) differentiated by the composition of the first coordination sphere: Cys5, Cys8, Cys26, and Cys29 (ZF1); Cys16, Cys19, His39, and His33 (ZF2); Cys55, Cys50, Cys72, and His75 (ZF3).

The ZBD model employed in the present study was extrapolated from the pdb entry 6zsl.^12^ This domain can be easily identified as the N-terminal segment comprising the 2–111 residues, made of 10 β-sheet strands (S1–S10) and two α-helices (H1–H2) (Figure 1). The H2 domain ensures the anchoring of the ZBD to the remaining portions of the nsp13 through the formation of three helices bundled with HA (117–124) and HB (128–150), hence, we also included two helices HA and HB (truncated at 146) into our ZBD model (Figure 1).

**Figure 2:**
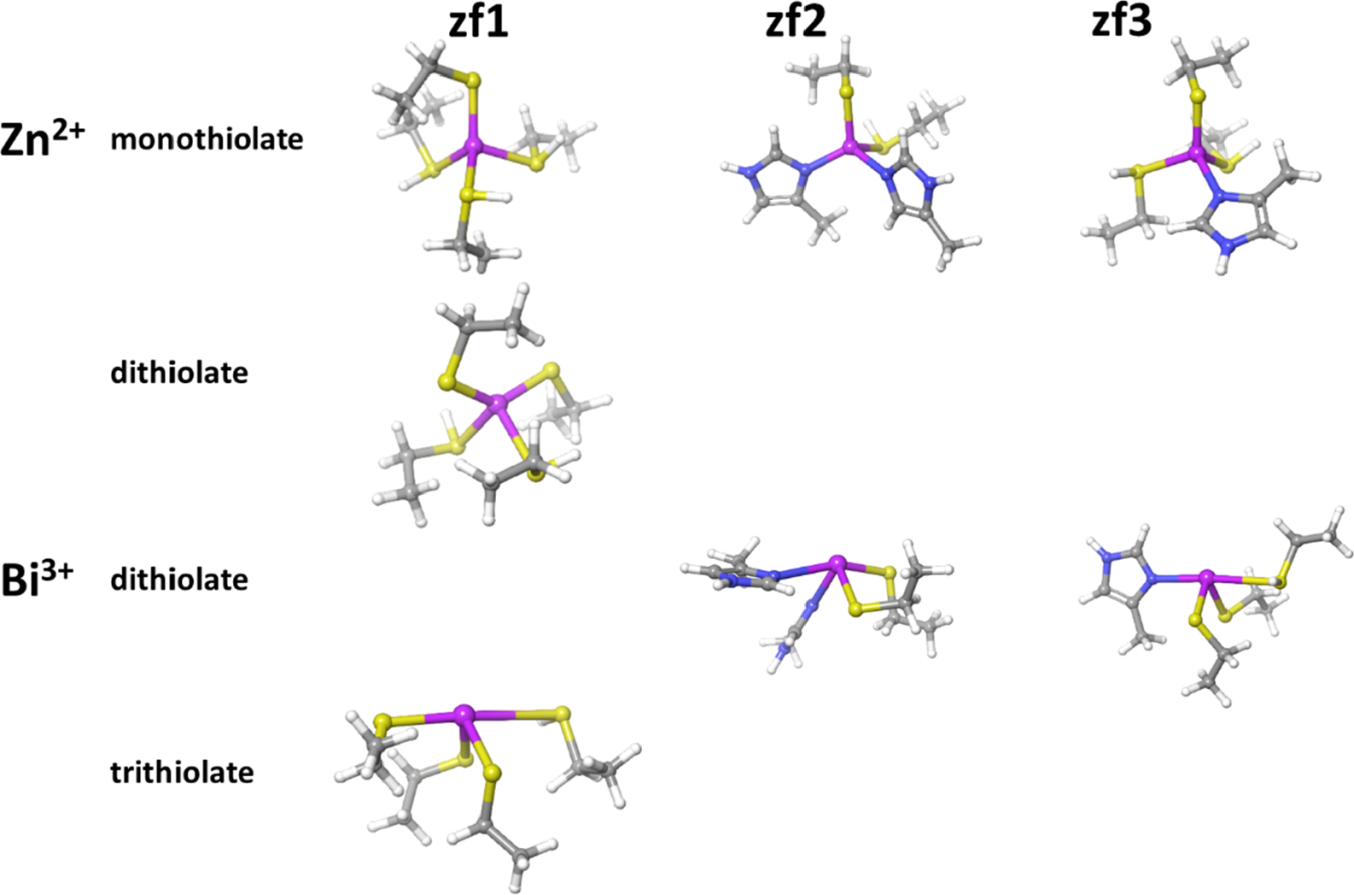
Rendition of the DFT-optimized structures for models of the zinc-finger domains coordinating either Zn^2+^ or Bi^3+^ in their most stable protonation states.

Preliminarily, the ZF sites were investigated by means of DFT approaches. For this purpose, simplified models of the metal-binding sites were obtained by replacing the coordinating amino acids with ethylthiol or ethylthiolate for cysteine in its neutral or deprotonated forms and the 1- or 2-methylimidazoles for histidine coordinated with either delta or epsilon nitrogen, respectively (Figure 2).

The cysteines which are included in the structures of ZFs can be either in the neutral or a deprotonated form, depending on the pH of the biological milieu. The presence of multiple cysteines in the studied ZFs yields the possibility of several states of protonation. Thus, the finger ZF1 can be deprotonated up to four times since it consists of four cysteine residues, whereas the corresponding values for ZF2 and ZF3 are two and three possible deprotonations due to the coordination spheres with two and three cysteines, respectively.

### Stability of ZFs with and without Metals

To assess the stability of ZFs ZF1, ZF2, and ZF3 in their either apo or metal-bound states and as a function of their ionization state, we exploited the calculation of the residue interaction energies affecting all possible protomeric forms of ZF1, ZF2, and ZF3. This energy parameter was simply calculated as the difference between the energy of either whole or apo (without metal) forms of each ZF model and the sum of the single-residue energies; for instance, the residue interaction energy for the whole ZF3 model with two deprotonated and one neutral cysteine is calculated as the energy for the reaction
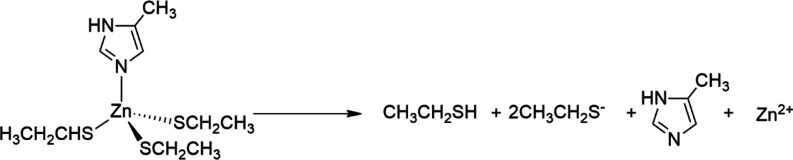


The coordination spheres of both zinc- and bismuth-containing structures were found to display similar values of residue interaction energies for their apo models (Table S1); hence, for the sake of simplicity, we aggregated the zinc and bismuth data by reporting the average values of apo residue interaction energies (Table 1). This analysis showed that the energetic instability of the ZFD increases with the number of deprotonated ethylthiolate species, and it can be explained based on the mutual electrostatic repulsion affecting the negatively charged ethylthiolates, for example, when the number of thiolates increases progressively from one to four, the residue interaction energy increases steeply in the trend 4.2, 22.6, 52.8, and 116.8 kcal/mol, respectively. Analogous trends were found for ZF2 and ZF3, although the corresponding dissociation energies for complete deprotonation were only 41.9 and 71.9 kcal/mol, respectively. It is peculiar to notice that the singly deprotonated forms of both ZF1 and ZF3 are slightly less stable than the neutral form, by 2.0 and 0.2 kcal/mol, respectively; these data are probably ascribable to the polar interactions occurring between protonated and deprotonated thiols that partially fade the mutual electrostatic repulsion of the ligand atoms. Thus, our calculations allowed us to infer that the most stable apo forms of ZF1 and ZF3 are singly deprotonated, while those of ZF2 are neutral.

**Table 1:** Residue Interaction Energies Calculated in Zinc-Finger Domains ZF1-3 With (Metal-Bound) and Without (Apo) the Metal Center and at Different Protomeric States of the Coordinated Thiols: N = 4-Methyl Imidazole, SH = ethylThiol, S^–^ = Ethylthiolate.^a^

domain	metal coordination	apo	metal-bound	
			Zn^2+^	Bi^3+^
ZF1	(SH)_4_	6.3	–89.2	–235.1
	(S^–^) (SH)_3_	4.2	–180.3	–335.3
	(S^–^)_2_(SH)_2_	22.6	–228.7	–445.8
	(S^–^)_3_(SH)	52.8	–260.0	–504.6
	(S^–^)_4_	116.8	–244.9	–520.2
ZF2	N_2_(SH)_2_	10.8	–146.2	–291.0
	N_2_(S^–^) (SH)	16.3	–210.2	–401.8
	N_2_(S^–^)_2_	41.9	–249.7	–468.7
ZF3	N(SH)_3_	12.2	–108.6	–266.5
	N(S^–^) (SH)_2_	12.0	–191.9	–368.3
	N(S^–^)_2_(SH)	35.2	–237.4	–461.2
	N(S^–^)_3_	71.9	–258.8	–507.6

a All values are in kcal/mol.

As expected, with the binding of the metal cations, Zn^2+^ and Bi^3+^, the stability of zinc-finger complexes increases drastically due to an overall favorable electrostatic attraction between the metal center and ligands, especially the negatively charged ethylthiolates, and due to the presence of coordinative bonds (Table 1). Moreover, the greater positive charge at Bi^3+^ explains the overall higher stability of Bi-based ZF complexes with respect to the Zn-based complexes, being almost twice higher in all cases. In the case of Zn^2+^, the most stable complexes are formed with ZF1 and ZF3, with three ethylthiolates in each case, with dissociation energies of about 260 kcal/mol, and two ethylthiolates with ZF2 (Table 1). The most stable complexes of Bi^3+^ are always those with the higher number of ethylthiolates, disclosing dissociation energies in the range 470–520 kcal/mol, in which a strong electrostatic stabilization is achieved.

An assessment of the protonation state of the ZF domains, when either Zn^2+^ or Bi^3+^ is coordinated at one ZF domain, was performed by the calculation of the decomplexation and deprotonation free energies, by using the thermodynamic cycle depicted in the Scheme 1.

By using this scheme, we expressed the deprotonation free energies of metal-bound complexes, Δ*G*^0^_deprot_^(M)^ (Table 2), in terms of the deprotonation free energy of the corresponding apo structure, Δ*G*^0^_deprot_^()^, and from the difference between the metal decomplexation free energies in the two evaluated protonation states (Scheme 1). We thus obtained a clear statement of the protonation state of each ZF site upon coordination with either Zn^2+^ or Bi^3+^ (Table 2). The Zn^2+^ coordination at ZF1 favors the monothiolate form, as indicated by the negative free energy for the deprotonation of the all-thiol model, in equilibrium with some smaller amount of the dithiolate form, whereas the Bi^3+^ coordination favors the trithiolate form of ZF1. On the other hand, both ZF2 and ZF3 structures assume the monothiolate or dithiolate protonation states upon coordination of Zn^2+^ or Bi^3+^, respectively (Figure 2).

**Table 2:** Deprotonation Free Energies (Δ*G*^0^_deprot_^(M)^) for Zinc-Finger Domains Complexed With Zn^2+^ or Bi^3+^ and Estimated in Terms of the Deprotonation Free Energy of the Unbound ZFD and Decomplexation Free Energies via the Thermodynamic Cycle of Scheme 1.^a^

domain	metal coordination	Δ*G*^0^_deprot_^(M)^	
		Zn^2+^	Bi^3+^
ZF1	(SH)_4_	–37.2	–52.4
	(S^–^) (SH)_3_	0.6	–57.6
	(S^–^)_2_(SH)_2_	24.6	–12.8
	(S^–^)_3_(SH)	55.6	45.7
ZF2	N_2_(SH)_2_	–13.4	–59.5
	N_2_(S^–^) (SH)	10.8	–15.3
ZF3	N(SH)_3_	–24.7	–58.6
	N(S^–^) (SH)_2_	5.8	–42.3
	N(S^–^)_2_(SH)	26.0	8.0

a The different protomeric states of the coordinated thiols: N = 4-methyl imidazole, SH = ethylthiol, S^–^ = ethylthiolate. All values are in kcal/mol.

The calculated geometries of the Zn^2+^ and Bi^3+^ complexes with the models of ZF domains in their most stable protonation states are reported in Figure 2, while the coordinative bond distances are collected in Table 3. These models were first employed to assign the protonation states to the coordinative side chains forming the ZF1, ZF2, and ZF3 sites of nsp13 ZBD. As a methodological criterion to identify neutral and deprotonated Cys side chains, we superimposed the DFT-optimized models of zinc-finger domains over the respective sites and consistently annotated the cysteine residues to be considered deprotonated with the Cym label (Figure 1). As expected, independently on the bound metal, thiolate groups form shorter coordinative bonds compared to thiols, with a length decrease of 0.3–0.5 Å (Table 3). Therefore, the coordinative bonds formed by Bi^3+^ are longer by 0.3–0.6 Å than those formed by Zn^2+^; these data indicate that replacement of Zn^2+^ with Bi^3+^ may cause an increase in the mutual distances between the nsp13 residues forming the ZF motifs. On the other hand, an even more drastic difference in the coordination geometries of Zn^2+^ and Bi^3+^ complexes is clearly appreciable: all Zn^2+^ complexes assume the symmetrical, tetrahedral geometry, whereas Bi^3+^ complexes adopt the hemidirected distorted tetrahedral geometry in any case. This latter geometry is determined from the presence of the 6s^2^ electron pair on the Bi^3+^ metal center and is expected to exert a marked distortion of the nsp13 backbone because it causes drastic displacement of the coordinative ligands compared to their placement in the respective Zn^2+^ complexes (Figure 1).

**Table 3:** Average Bond Lengths in Zinc-Finger Domains With Zn^2+^ and Bi^3+^^b^

domain	coordination	average bond length					
		Zn–SH	Zn–S^–^	Bi–SH	Bi–S^–^	Zn–N	Bi–N
ZF1	(SH)_4_	2.55		2.80			
	(S^–^) (SH)_3_	2.50	2.23	2.87	2.53		
	(S^–^)_2_(SH)_2_	2.61	2.26	2.86,3.40^a^	2.51		
	(S^–^)_3_(SH)	4.3^a^	2.28	3.48	2.57		
	(S^–^)_4_		2.39		2.68		
ZF2	N_2_(SH)_2_	2.46		2.91		2.01	2.25
	N_2_(S^–^) (SH)	2.54	2.27	3.05	2.51	2.04	2.33
	N_2_(S^–^)_2_		2.31		2.56	2.10	2.51
ZF3	N(SH)_3_	2.46		2.83		2.00	2.24
	N(S^–^) (SH)_2_	2.55	2.23	2.86	2.49	2.03	2.42
	N(S^–^)_2_(SH)	2.67	2.28	3.11	2.52	2.07	2.41
	N(S^–^)_3_		2.35		2.57	2.14	2.79

a No covalent bond between sulfur and the metal center.

b All values are in angstroms.

Other structural modulations, occurring as a consequence of the Zn^2+^-to-Bi^3+^ exchange, were unveiled by the analysis of the atomic charges in the examined metal complexes of ZF1, ZF2, and ZF3, as reported in Table 4. As expected, the NBO charge on the Zn^2+^ metal center was found to be lower compared to that on Bi^3+^, although by only 0.3–0.4 charge units. On the other hand, the atomic charges on metal-bound atoms S and N were found to assume similar values, with differences in the order of 0.2 or less units of charge. Interestingly, NBO charge of the S atom of thiolate and thiol ligands showed a different modulation: while coordination at Zn^2+^ or Bi^3+^ induces almost similar charges on thiol sulfurs, the S atoms of thiolates bound to Zn^2+^ were found to be slightly more negative compared to Bi^3+^ (Table 4).

**Table 4:** Average Atomic Charges in Zinc Fingers With Zn^2+^ and Bi^3+^. All Values in e^-^ Units.

domain	metal coordination	average charges							
		Zn-bound				Bi-bound			
		Zn	SH	S^–^	N	Bi	SH	S^–^	N
ZF1	(SH)_4_	0.72	–0.10			1.00	–0.04		
	(S^–^) (SH)_3_	0.55	–0.13	–0.42		0.93	–0.13	–0.24	
	(S^–^)_2_(SH)_2_	0.42	–0.18	–0.46		0.94	–0.19	–0.34	
	(S^–^)_3_(SH)	0.52	–0.28	–0.52		0.86	–0.24	–0.41	
	(S^–^)_4_	0.42		–0.60		0.86		–0.51	
ZF2	N_2_(SH)_2_	0.38	–0.11		–0.16	1.21	–0.14		–0.31
	N_2_(S^–^) (SH)	0.36	–0.16	–0.46	–0.18	1.11	–0.18	–0.31	–0.32
	N_2_(S^–^)_2_	0.36		–0.54	–0.18	1.02		–0.40	–0.37
ZF3	N(SH)_3_	0.47	–0.10		–0.18	1.14	–0.07		–0.29
	N(S^–^) (SH)_2_	0.42	–0.15	–0.41	–0.20	1.06	–0.10	–0.25	–0.42
	N(S^–^)_2_(SH)	0.36	–0.17	–0.49	–0.20	0.95	–0.23	–0.34	–0.38
	N(S^–^)_3_	0.33		–0.56	–0.20	0.90		–0.42	–0.44

### MD Simulations

MD simulations were thus performed to model the structure of either the Zn^2+^- or Bi^3+^-bound ZBD of nsp13, in particular to unveil how the metal exchange may affect the molecular interaction properties of such an important domain. The preliminary DFT investigation of the reduced models of ZF1, ZF2, and ZF3 yielded important structural insights into the structure of the nsp13 ZBD. In particular, the protonation states of the three metal binding sites coordinated with either Zn^2+^ or Bi^3+^ were determined, thus allowing us to correctly model the Zn-bound and the Bi-bound ZBD models. Therefore, the optimized geometries of the reduced models of ZF1, ZF2, and ZF3 demonstrated that Bi^3+^ compared to the Zn^2+^ metal center requires rather different parametrization: besides the obvious differences in the coordination distances, the Bi-bound ZF sites present a hemidirected geometry due to the presence of the 6s lone pair.

**Figure 3:**
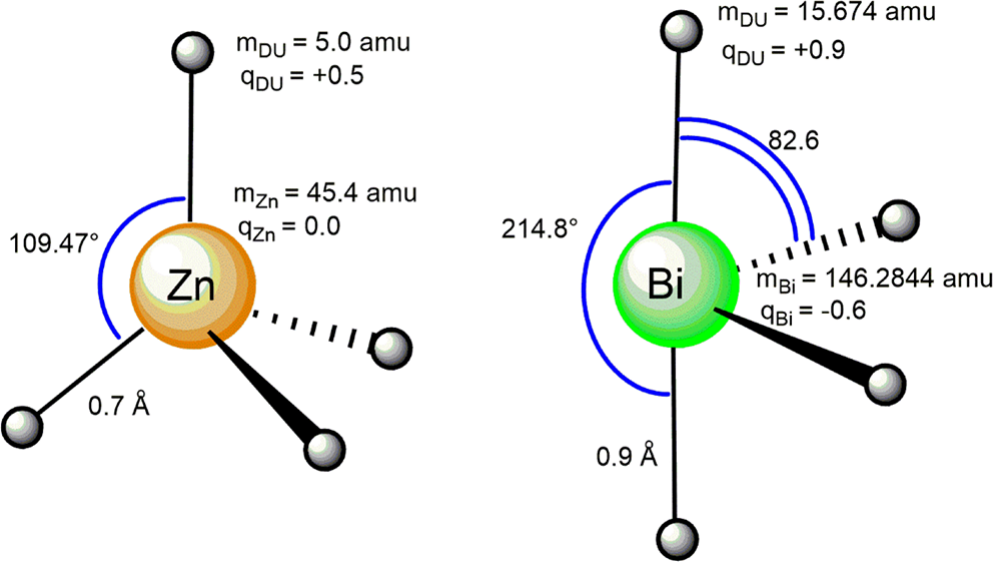
Cationic dummy atom models of Zn^2+^ (left) and Bi^3+^ (right). Masses are in amu, distances are in Å, angles are in degrees, and charges are in e^–^ units.

Based on the DFT outcomes, the ZF1, ZF2, and ZF3 metal centers were described using the cationic dummy atom (CDA) parameterization scheme that allows to better account for the coordination geometry, as already shown elsewhere.^40^ The CDA models used to describe the Zn^2+^ and Bi^3+^ metal centers are depicted in Figure 3.

The fractional partition of both metal masses and charges among the cationic dummy atoms (Du) and the values of distances and angles were extrapolated from the CDA model previously adopted to describe either tetrahedral or hemidirected geometry of the Pb^2+^ ion.^40^ Based on the higher atomic radius of Bi compared to that of Zn, the Bi-Du distances were assumed to be longer by 0.2 Å. Noteworthy, the negative charge on the Bi^3+^ metal center was employed to account for the electron density ascribed to the 6s^2^ lone pair. Within these methodological choices, the two metal centers are correctly differentiated in terms of (i) directionality of the coordinative interactions, (ii) charge distribution, and (iii) size.

**Figure 4:**
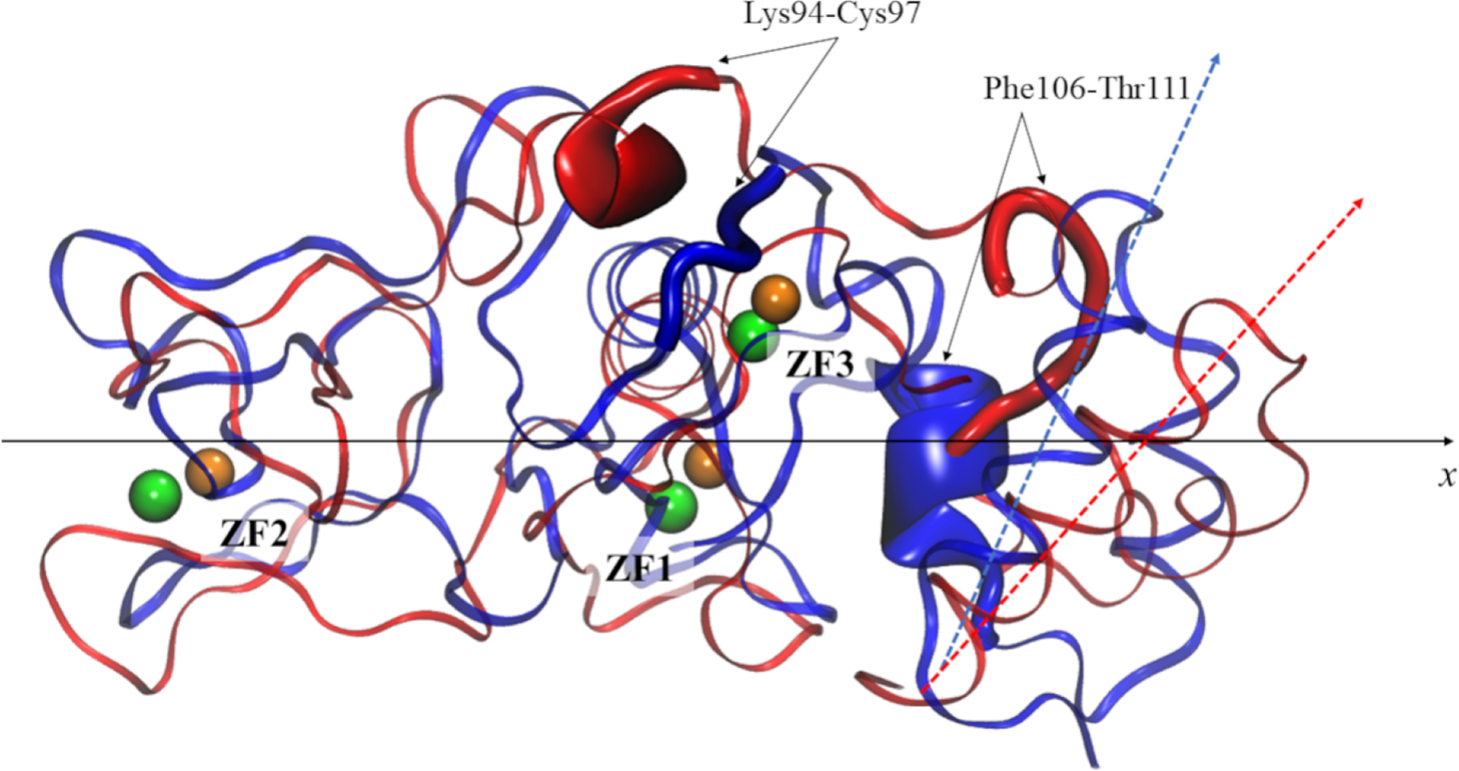
Steric reshaping of the ZBD of nsp13 induced by the Zn^2+^/Bi^3+^ exchange. The models of Zn-bound (blue) and Bi-bound (red) ZBDs of nsp13 are depicted as ribbons. The Zn^2+^ and Bi^3+^ ions are shown as orange and green spheres, respectively. The ZBD segments featuring massive reshaping with the Zn^2+^/Bi^3+^ exchange are labeled and shown as magnified cartoon models. The slight torsion (with respect to the *x* axis) of the H2|HA|HB bundles of Zn-bound and Bi-bound models is exemplified by the orientation of the HA domains, blue and red dashed arrows, respectively.

The calculated trajectories of Zn-bound and Bi-bound ZBDs of nsp13 were found to gain equilibration in 5 ns and 155 ns, respectively, thus yielding 45 ns of stable trajectory, as detectable from the backbone RMSD profiles. As expected, the Bi-bound system trajectory was characterized by a longer lag time to equilibration because of the more extensive conformational rearrangement of the ZBD backbone (Figure S2). These data evidenced that the steric and electrostatic deviations induced by Bi^3+^ in place of Zn^2+^ metal centers may potentially alter the shape and the anchoring of the nsp13 ZBD. Indeed, radial distribution function analyses of the Zn-bound and Bi-bound trajectories showed how the coordination of Bi^3+^ in place of Zn^2+^ slightly increased both metal–ligand and metal–metal distances (Figure S3). The peptide folding of the nsp13 ZBD domain detected in both Zn-bound and Bi-bound snapshots is almost superimposable to the X-ray model of the 2–111 segment. On the other hand, extensive deviations of the protein fold were detected in the C-terminal portion 112–145 of the Bi-bound system, corresponding to a torsion of the H2|HA|HB bundle with respect to the major axis of the ZBD (Figure 4).

The most representative conformations of Zn-bound and Bi-bound ZBDs, extracted from the corresponding trajectories, are reported in Figure S4. It is noteworthy how the ZBD topology is not significantly affected by the Bi^3+^/Zn^2+^ exchange, and the mutual positions of ZF1, ZF2, and ZF3 were only marginally affected. Indeed, the secondary structure analysis, performed on the X-ray structure and on the most representative conformations, confirmed that a slight steric reshaping occurred in the Bi-bound ZBD compared to the Zn-bound ZBD model. More specifically, besides the loss of the short S5 strand (both Zn- and Bi-bound) and the S1/S2 (only Bi-bound) structures (Table S2) , a higher reshaping was detected in the 94–97 segment (α-helix only for the Bi-bound model) and in the 106–111 segment (α-helix only for the Zn-bound model) (Figure 4, Table S2).

### ATOMIF Analysis

To better assess the impact of Bi^3+^/Zn^2+^ exchange on the molecular interaction properties of the nsp13 ZBD, the electrostatic and hydrophobic molecular potentials were investigated by using the ATOMIF approach, developed and applied for the investigation of protein systems.^33,59−61^

The average molecular electrostatic potential of the Zn-bound and Bi-bound ZBD was calculated by using the APBS approach (see Methods) to better describe the fading effect of the bulk. Interestingly, the values of average MEP of the Bi-bound ZBD were found between 0 and +0.38, whereas the average MEP of the Zn-bound system were found in the −0.15–+0.30 range, thus evidencing how the Zn^2+^/Bi^3+^ exchange increased the positive character of the ZBD.

**Figure 5:**
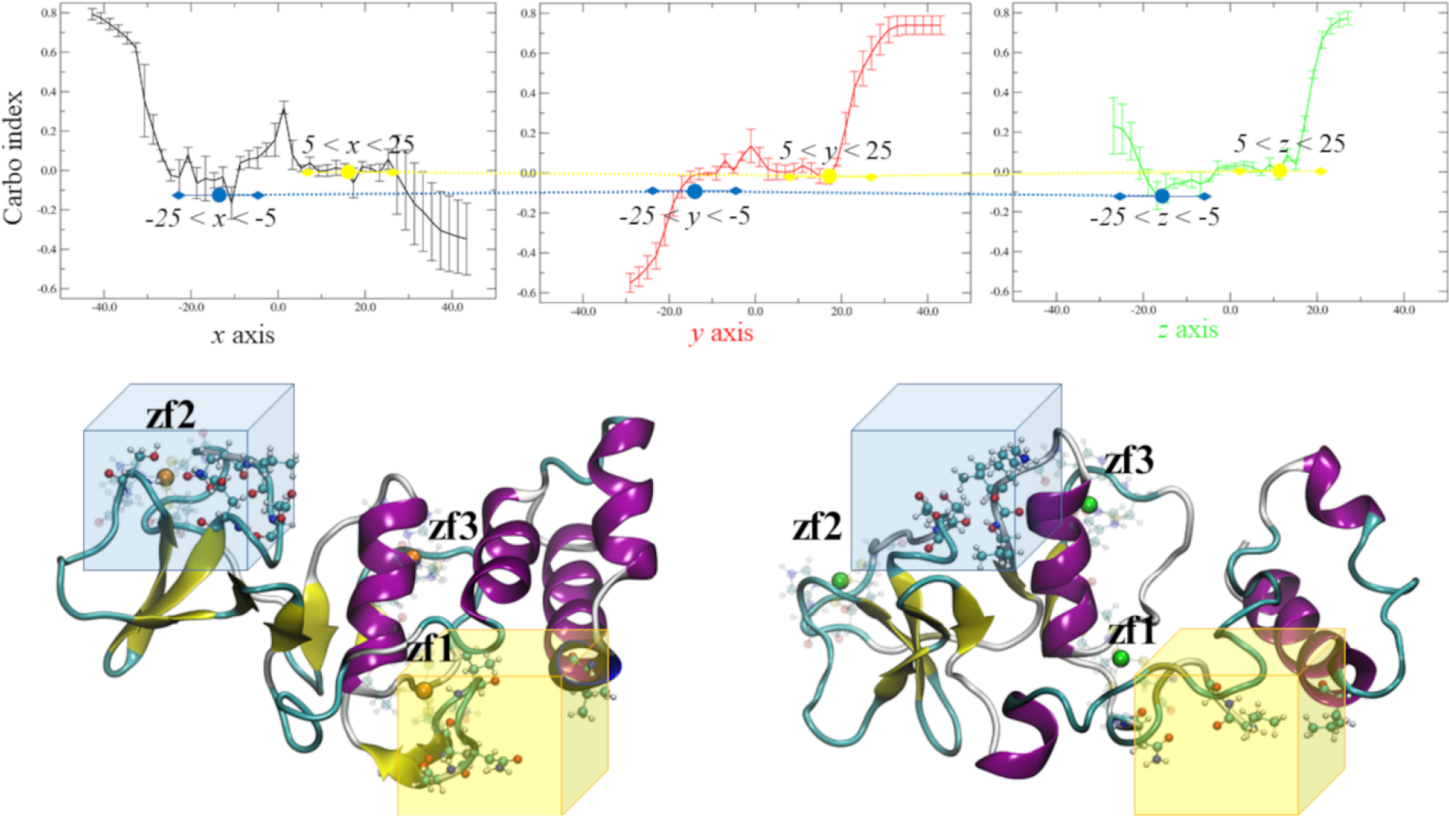
Carbo index profiles along *x*, *y*, and *z* axes, obtained by the comparison of APBS-MEP fields of the Zn-bound and Bi-bound nsp13 ZBD (top). Regions comprising a minimum of the CI profile are quoted. The regions of space with lower electrostatic similarity between Zn-bound (Zn = orange sphere and ZF residues = glassy) and Bi-bound (Bi = green sphere and ZF residues = glassy) models retrieved from the CI profiles were indicated (shaded cubes), and the protein residues included in these regions are shown (ball-and-stick representation).

The molecular electrostatic similarity between the Zn-bound and the Bi-bound models was then calculated in terms of the Carbo similarity index (CI) and elaborated by using the ATOMIF tool (Figure 5). More specifically, the three-dimensional MEP fields of two protein models were analyzed to scan the electrostatic similarity along the main inertial axes (*x*, *y*, and *z*) of the two protein models and yielded three CI profiles (Figure 5).

As shown, our calculations evidenced two regions of lower electrostatic similarity, one (Figure 5, yellow cube) placed on the N-terminus and partially involving the H2|HA|HB bundle that ensures the anchoring of the ZBD to the other nsp13 domains and the other (Figure 5, blue cube) either close to (Bi-bound) or including (Zn-bound) the ZF2 region. These two regions were identified by grouping the minima of the *x*, *y*, and *z* profiles with similar values of CI, that is, CI = 0.0 and CI = −0.1, respectively, and by assuming ±10 Å ranges for each dimension (Figure 5). In both cases, the MEP similarity lowering is paralleled by substantial conformational rearrangements. For example, the ZF2 domain of the Bi-bound ZBD was displaced by the backbone rearrangement occurring in the β-sheet bundle region, that is, S4–S10 (Figure 1), which changed the spatial disposition of the ionized residues compared to those of the Zn-bound ZBD. On the other hand, the rotation of the helices’ bundle around the major axis of the Bi-bound ZBD affected the interface with ZF1 and ZF3 regions, and caused a substantial conformational variation of the N-terminus compared to that of the Zn-bound ZBD.

Similarly, changes in the hydrophobic features of the nsp13 ZBD, in response to the Bi/Zn exchange, were investigated using the ATOMIF tool. The representative structures of Zn-bound and Bi-bound ZBDs were mapped in the same 3D grid by using the DRY probe, which is responsive to the hydrophobic effect.

**Figure 6:**
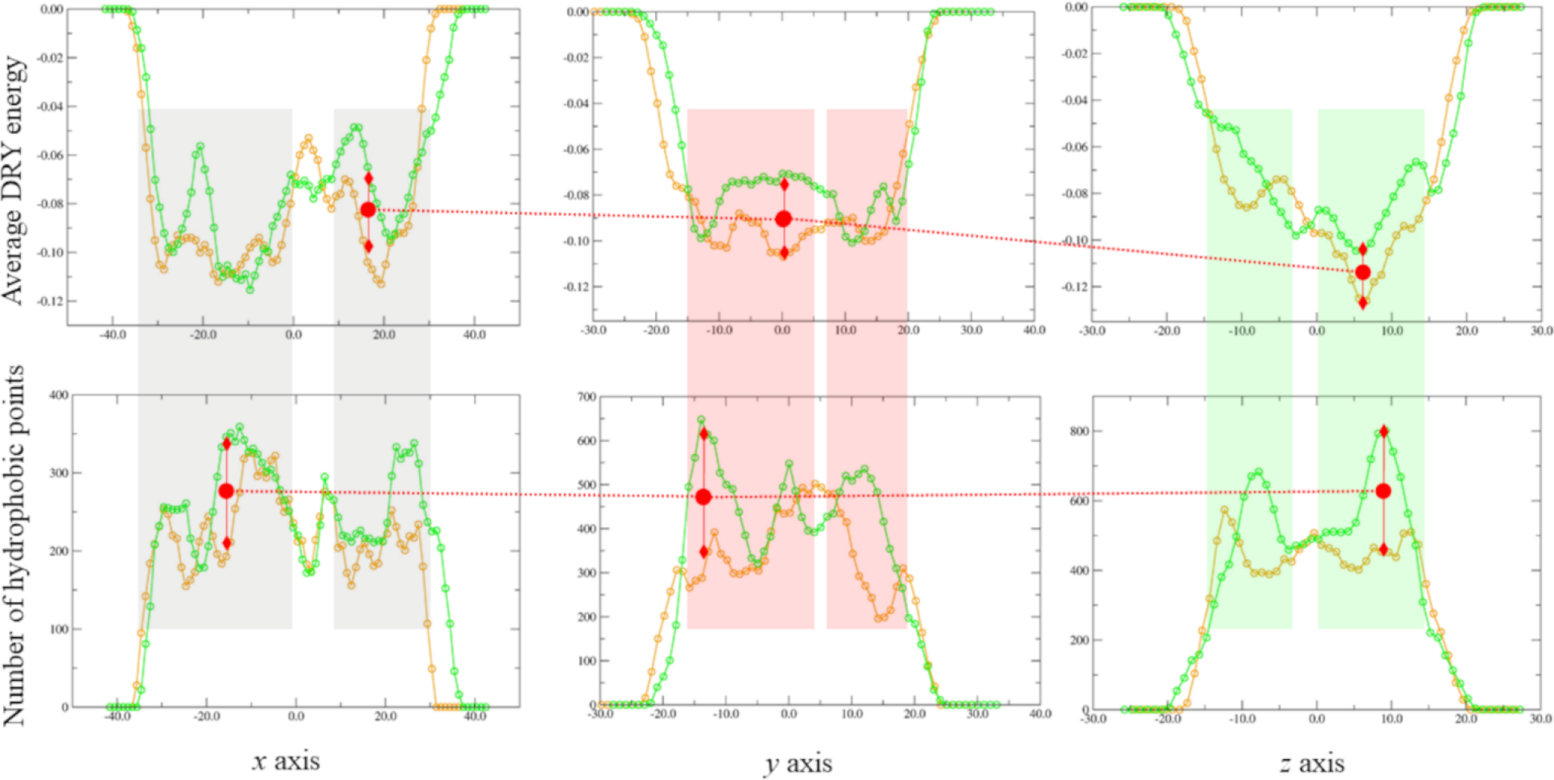
Profiles of average hydrophobic field intensity (HFI) and the number of hydrophobic points (#HF) along *x*, *y*, and *z* axes for the Zn-bound (orange) and Bi-bound (green) nsp13 ZBD. Regions comprising either minima of the HFI profile or maxima of the #HF profile are shaded. Two regions displaying pronounced variations of either HFI or #HF profile were annotated (red dashed arrows).

**Figure 7:**
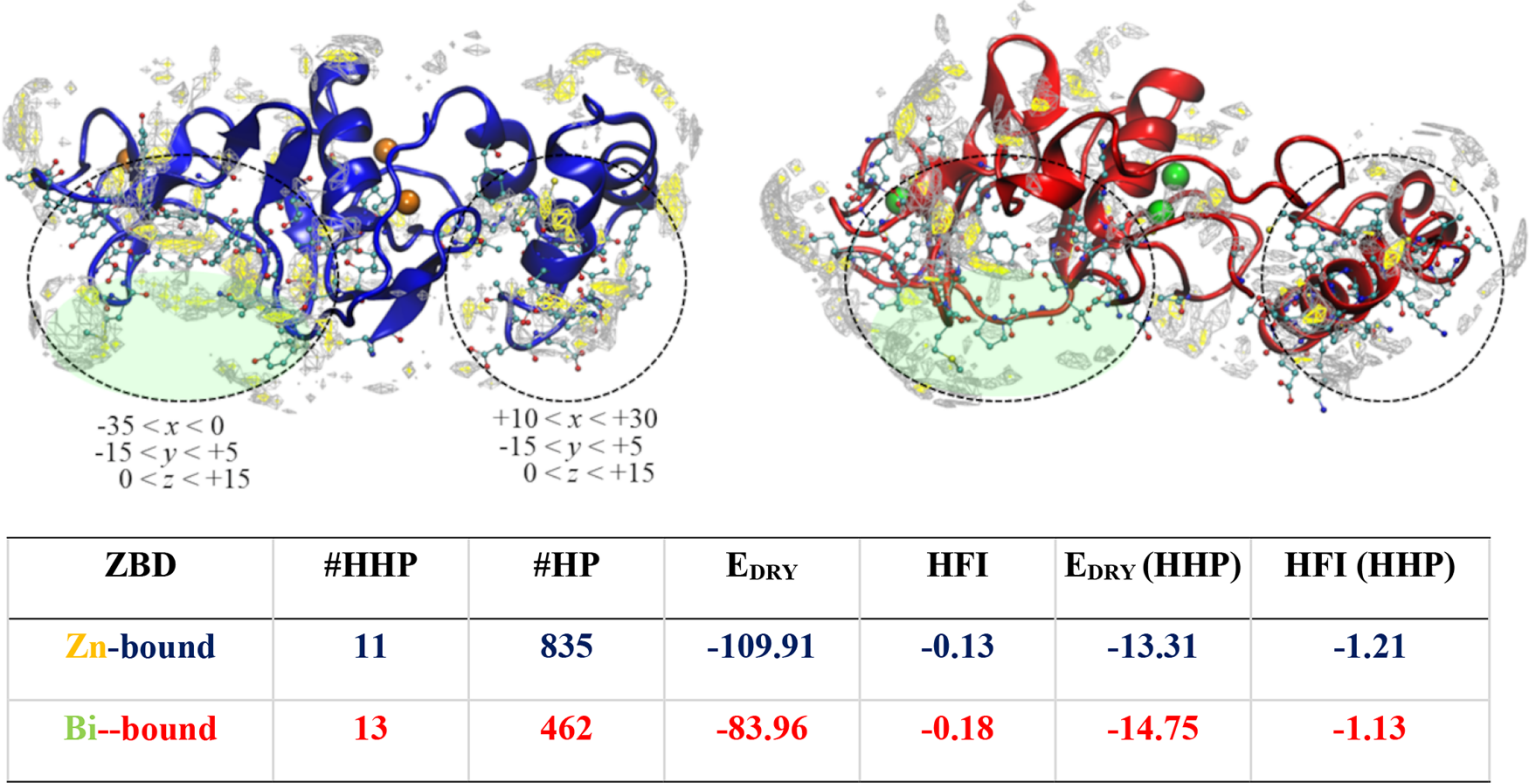
Top: rendition of the hydrophobic field of Zn-bound (blue cartoon) and Bi-bound (red cartoon) models: DRY = −0.4 (silver mesh) and DRY = −0.9 (yellow mesh) isosurfaces are depicted. Two regions obtained by combining the shaded regions of HFI profiles (Figure 6) were delimited by dashed circles, and the relative coordinate ranges are also reported (values in Å). Table: HFI analysis of the region −30 < *x* < −10, −10 < *y* < 10, and 0 < *z* < 10 (green shade): #HHP = number of highly hydrophobic points, #HP = number of hydrophobic points, *E*_DRY_ = sum of the DRY interaction energies, HFI = average DRY energy, E_DRY_(HHP) = sum of the DRY energies of HHP, and HFI (HHP) = average DRY energy of HHP. All energy values are in kcal/mol.

Again, the 3D maps (i.e., the MIF) of the DRY interaction with either Zn-bound or Bi-bound ZBDs were analyzed along the three principal axes of inertia in terms of the total DRY energy, weighted with the number of non-zero points, which is a measure of the hydrophobic field intensity (HFI) within each grid slice (see Methods for details). The corresponding HFI profiles were reported in Figure 6. The highlighted ranges correspond to the regions of space, featured by stronger DRY fields, and thus ascribable to higher extents of exposed hydrophobic residues. These regions were in particular characterized by the presence of minima identified in both Zn-bound and Bi-bound models (Figure 6) and give rise to coordinate ranges delimiting the regions of space with higher intensities of the DRY field, that is, higher hydrophobicity. The graphical inspection of these coordinate ranges allowed to identify two regions of hydrophobicity (Figure 7). The wider hydrophobic region was identified in proximity to the ZF2 site and was predominantly delimited by the S3–S10 strands and their interconnecting loops (Figure 7).

As depicted in Figure 7, this region disclosed appreciable rearrangements of the Bi-bound ZBD backbone compared to the Zn-bound model; in particular, we detected the significant displacement of the 66–69 segment that basically reshapes a wide portion of the cleft vicinal to the ZF2 domain (Figure 7).

This sub-region, delimited by the ranges −30 < *x* < −10, −10 < *y* < 10, and 0 < *z* < 10, is characterized by a peculiar hydrophobic exposure in the Zn-bound ZBD that is expected to be functionally relevant due to its involvement in the interaction with nsp12 (vide infra). Interestingly, while the number and the DRY energy of the highly hydrophobic points (HHPs) in this sub-region were found to be similar in either Zn-bound and Bi-bound ZBDs, the higher number of hydrophobic points (HPs) and almost twofold DRY energy (E_DRY_) of the Zn-bound model evidenced the lesser extension of the hydrophobic portions in the Bi-bound model compared to that in the Zn-bound model (Figure 7, table). Again, these data were ascribed to the backbone repositioning occurring as part of the steric reshaping of the ZBD of nsp13 in concomitance with the Zn^2+^/Bi^3+^ exchange.

## Discussion and Conclusions

Several millions of deaths and economic decline due to worldwide lockdowns caused by multiple waves of SARS-CoV-2 variants in 2021–2022 are the reasons for an unprecedented research effort by the scientists from all over the world to find an efficient therapeutic agent which could robustly interfere in the viral machinery of this disease, inhibiting its development. The identification of molecular components of the SARS-CoV-2 particle has prompted the development of vaccines and immunotherapy protocols able to counteract the dramatic impact of the sadly known COVID19 pandemic. However, the high mutational rate of this virus together with its wideworld diffusion favors the appearance of variants against which the developed vaccine or immune therapies may result to be less effective or even inadequate. In this view, the search for chemical compounds that may hamper transmission, propagation, and harmful potential of the SARS-CoV-2 infection by destabilizing the molecular components of the viral machinery and, more importantly, may maintain their antiviral potential against possibly all variants, should be considered a further indispensable target. In this frame, the activity of metal compounds, including either transition metal or metalloid complexes, has been recently explored in vitro to evaluate their possible repurposing in the anti-SARS-CoV-2 therapy, based on their selective binding at protein nucleophile sites, such as selenocysteine or cysteine side chain groups.^62^ A key advantage in the development of metallic therapeutics for the treatment of the SARS-CoV-2 infection is represented by their ability to bind on single and often functionally crucial protein residues, and thus, their efficacy is expected to be less liable to the occurrence of new viral variants.^63^ The targeting of invariant components of the SARS-CoV-2 molecular equipment represents a valuable strategy for the development of antiviral therapeutics. In this respect, we envision that the viral proteins featured by the presence of metal-binding domains may be also particularly suitable targets because of the high chemoselectivity intrinsic to metal coordination constructs. Among the functional proteins of the SARS-CoV-2 virus, the zinc-dependent nsp13 is a helicase that acts as a major component of the viral infection mechanism.^12^ Indeed, the interaction of nsp13 with the RNA-dependent RNA polymerase holoenzyme (RdRp) is essential to warrant the replication of the viral genome and, in concert with other proteins, may suppress the interferon production and signaling in response to the SARS-CoV-2 infection.^5,8^ The nsp13 protein is characterized by an N-terminal ZBD made up of three zinc-finger motifs, ZF1-3, anchored to the overall protein structure through a bundle of three α helices (Figures 1 and S1). Several structural evidences have shown that the ZBD of nsp13 is involved in interaction with nsp12 in the multifunctional protein assembly forming the RdRp.^22,23,29^ Although the disruption of the Zn^2+^ coordination may expectedly alter its structural stability and, in turn, induce an advantageous impairment of the nsp13 functionality, the ZF1-3 sites are located at the core of the ZBD, thus making the sequestration of Zn^2+^ ions, for instance, via chelation, particularly challenging.

An alternative and more viable approach is represented by the exchange of Zn^2+^ ions with a different metal cation, as reported by Yuan et al.,^25^ where it was shown that the administration of RBC resulted in exchange of Zn^2+^ ions with Bi^3+^, thus disrupting the nsp13 function. Assuming that the obstruction of nsp13 originates from the altered protein structure caused by the coordination of another metal, we analyzed the replacement of Zn^2+^ with Bi^3+^ ions in the ZF1-3 sites and its consequences on the shape and interaction features of nsp13 by means of multilayered computational approaches. The selection of Bi^3+^ ion as the metallic agent replacing Zn^2+^ from nsp13 was reputed advantageous, based on three important features: (i) Bi^3+^ salts have been long used as antimicrobial therapeutics, and thus, medicinal formulations of these agents are already available in the market,^64,65^ (ii) the higher formal charge compared to Zn^2+^ may favor electrostatically the insertion of Bi^3+^ at the ZF1-3 sites, and (iii) the Bi^3+^ ion has a bigger valence shell, compared to that of Zn^2+^ and is characterized by the presence of a 6s^2^ lone pair that favors the hemidirected coordination of this metal center. Density functional calculations confirmed that the Zn^2+^ to Bi^3+^ exchange leads to substantial changes in the geometrical and ionization properties of reduced models of ZF1-3. In fact, as summarized in Figure 2, the Bi^3+^ coordination compared to Zn^2+^ induces the deprotonation of one more thiol ligand, thus determining the same total charge in the ZF1-3 models. On the other hand, the Bi^3+^ metal center assumes the hemidirected geometry that, compared to the X-ray-detected tetrahedral coordination of Zn^2+^ ions, corresponds to a substantial geometrical reshaping of the ZF1-3 sites of the ZBD. To better assess the structural implications of such a ZF1-3 reshaping for the molecular interaction properties of the ZBD of nsp13, a combined molecular dynamics/molecular interaction field study was performed by using the ATOMIF tool. The structure of the ZBD bound to either Zn^2+^ or Bi^3+^, by assuming the protonation states of ZF1-3 sites determined by DFT calculations, was analyzed in order to extrapolate the most representative protein conformations. The CDA topology scheme was employed to describe the metal coordination at the ZF1-3 sites, mostly based on a CDA scheme previously probed by us.^40^ Our data evidenced slight changes in the overall ZBD fold upon Zn^2+^/Bi^3+^ exchange, although more appreciable reshaping was detected in proximity to the ZF2 site, segment 94–97, and in the H2|HA|HB bundle, represented by the loss of H2 in the Bi-bound model (Figure 4). The subsequent analysis of molecular electrostatic potential evidenced that the Zn^2+^/Bi^3+^ exchange induces changes in the ZBD electrostatics, again, in proximity of the ZF2 site and at the interface of the N-terminus with the helix bundle (Figure 5). On the other hand, the DRY field analysis allowed to identify a protein portion proximal to the ZF2 site, in which the extent of hydrophobic surface decreases when Bi^3+^ ions replace the Zn^2+^ ions.

**Figure 8:**
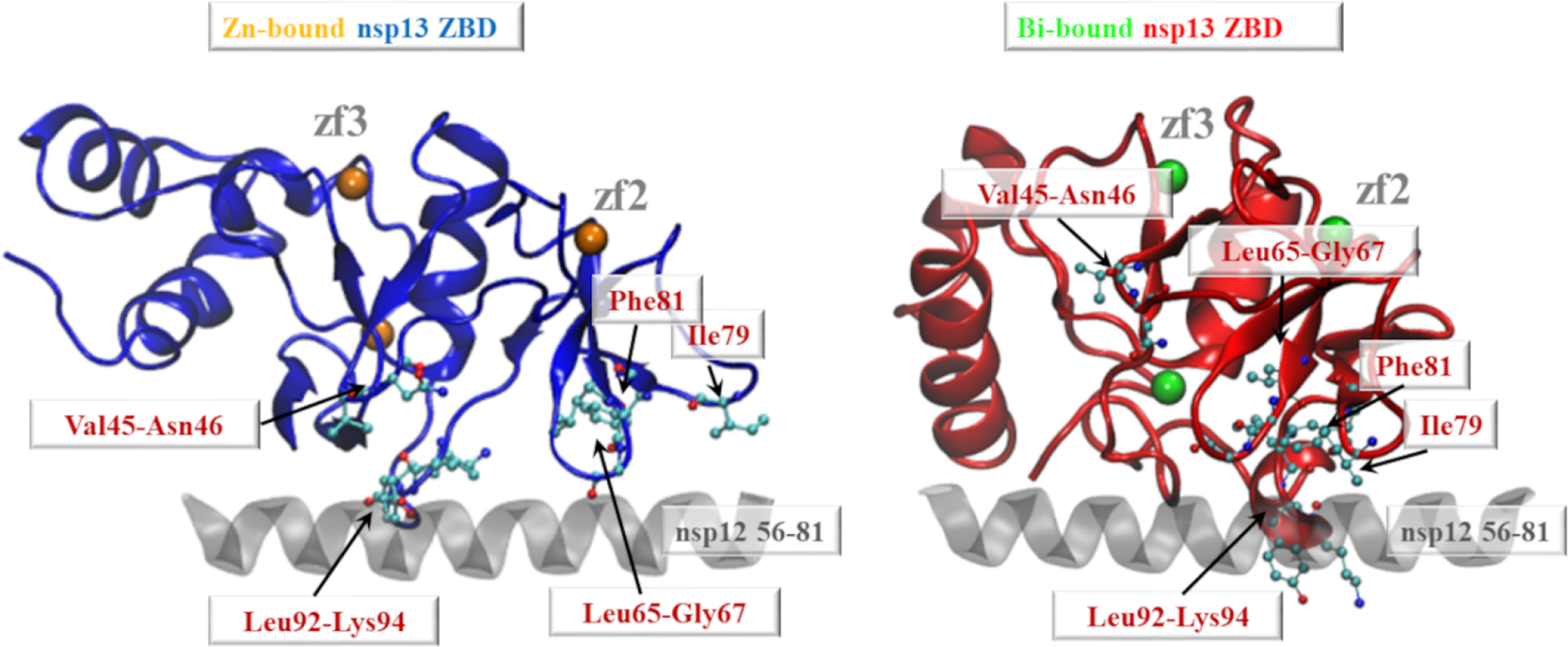
Rendition of the nsp13(ZBD)–nsp12(56–81) interaction obtained by superimposing the most representative MD conformations of the Zn-bound (left, Zn = orange sphere) and Bi-bound (right, Bi = green sphere) ZBD over the cryoelectron microscopy structure of the SARS-CoV-2 replication and transcription complex. For the sake of clarity, only the 56–81 segment of the nsp12 chain (gray cartoon) is shown. The nsp13 residues in the closest contact (at least one interatomic distance <3.5 Å) with the nsp12 cryoelectron microscopy model (pdb entry 7CXN^29^) were labeled and shown as a ball-and-stick representation.

The most important finding of this computational study is the identification of the tiled region with the nsp13 portion being involved in the interaction with the nsp12 protein. Indeed, both proteins are components of the SARS-CoV-2 replication and transcription complex, and their interaction is expected to be crucial to ensure the correct assembly of this multifunctional complex.^22,23,29^ The superimposition of the most representative MD conformations of the Zn-bound and Bi-bound ZBD over the homologous domain in the experimentally characterized SARS-CoV-2 replication and transcription complex allowed to precisely state that the hydrophobic pocket annotated through the ATOMIF analysis is the same that interacts with the 56–81 segment of the nsp12 component (Figure 8). Our analysis also showed that 10 over 12 close nsp13-nsp12 contacts detected experimentally were correctly predicted by our model of the Zn-bound ZBD, thus corroborating our modeling approach. In particular, the nsp12 fragment was found to interact with five portions of the ZBD of nsp13 (Figure 8, left). On the other hand, we found that the nsp12 fragment cannot be similarly hosted and that the residues involved in the nsp12 interaction of the Zn-bound ZBD presented markedly different positions in the Bi-bound ZBD model (Figure 8). We thus hypothesize that the ZBD reshaping induced by the Zn^2+^/Bi^3+^ exchange is able to cause both steric and hydrophobic alterations localized in the region of the nsp12 binding, consistently with a postulated weakening of the nsp13–nsp12 interaction.

Interestingly, the same portion of the N-terminal ZBD of nsp13 has been also identified by Armen et al.^22^ as a druggable, allosteric site that could be targeted by potential inhibitors of the nsp13:nsp12 assembly.

In conclusion, our calculations indicate that the replacement of Zn^2+^ ions with Bi^3+^ can fade the interaction between nsp13 and nsp12, thus showing a potential anti-SARS-CoV-2 activity. Besides the insights into the identification of an unselective antiviral agent, that is, the Bi^3+^ ion whose real effectiveness remains to be ascertained experimentally, we repute of extreme interest the possibility of modulating the molecular interaction features of a metal-binding protein, such as nsp13, by means of metal exchange. Therefore, our computational studies corroborate the importance of the hydrophobic region of the nsp13 ZBD involved in the binding of nsp12, already indicated by others,^22^ as a potential target site to be addressed in the seeking for novel anti-SARS-CoV-2 compounds.

## Supporting Information Available

Rendition of the X-ray structure of nsp13; RMSD and RMSF calculated from the Zn-bound and Bi-bound trajectories; DFT energy parameters calculated on both metal-bound and apo models of the ZF1-3 domains; and secondary structures of the nsp13 ZBD binding either Zn^2+^ or Bi^3+^ ions (PDF)

## References

[ref1] World Health Organization. WHO COVID-19 Dashboard. https://who.sprinklr.com/ (accessed April 10, 2022).

[ref2] Zhang L.; Lin D.; Sun X.; Curth U.; Drosten C.; Sauerhering L.; Becker S.; Rox K.; Hilgenfeld R. Crystal Structure of SARS-CoV-2 Main Protease Provides a Basis for Design of Improved a-Ketoamide Inhibitors. Science 2020, 368, 409–412. 10.1126/science.abb3405. 32198291 PMC7164518

[ref3] Jin Z.; Du X.; Xu Y.; Deng Y.; Liu M.; Zhao Y.; Zhang B.; Li X.; Zhang L.; Peng C.; Duan Y.; Yu J.; Wang L.; Yang K.; Liu F.; Jiang R.; Yang X.; You T.; Liu X.; Yang X.; Bai F.; Liu H.; Liu X.; Guddat L. W.; Xu W.; Xiao G.; Qin C.; Shi Z.; Jiang H.; Rao Z.; Yang H. Structure of Mpro from SARS-CoV-2 and Discovery of Its Inhibitors. Nature 2020, 582, 289–293. 10.1038/s41586-020-2223-y. 32272481

[ref4] Gao Y.; Yan L.; Huang Y.; Liu F.; Zhao Y.; Cao L.; Wang T.; Sun Q.; Ming Z.; Zhang L.; Ge J.; Zheng L.; Zhang Y.; Wang H.; Zhu Y.; Zhu C.; Hu T.; Hua T.; Zhang B.; Yang X.; Li J.; Yang H.; Liu Z.; Xu W.; Guddat L. W.; Wang Q.; Lou Z.; Rao Z. Structure of the RNA-Dependent RNA Polymerase from COVID-19 Virus. Science 2020, 368, 779–782. 10.1126/science.abb7498. 32277040 PMC7164392

[ref5] Yuen C. K.; Lam J. Y.; Wong W. M.; Mak L. F.; Wang X.; Chu H.; Cai J. P.; Jin D. Y.; To K. K. W.; Chan J. F. W.; Yuen K. Y.; Kok K. H. SARS-CoV-2 Nsp13, Nsp14, Nsp15 and Orf6 Function as Potent Interferon Antagonists. Emerg. Microbes Infect. 2020, 9, 1418–1428. 10.1080/22221751.2020.1780953. 32529952 PMC7473193

[ref6] Wrapp D.; Wang N.; Corbett K. S.; Goldsmith J. A.; Hsieh C.-L.; Abiona O.; Graham B. S.; McLellan J. S. Cryo-EM Structure of the 2019-NCoV Spike in the Prefusion Conformation. Science 2020, 367, 1260–1263. 10.1126/science.abb2507. 32075877 PMC7164637

[ref7] Lan J.; Ge J.; Yu J.; Shan S.; Zhou H.; Fan S.; Zhang Q.; Shi X.; Wang Q.; Zhang L.; Wang X. Structure of the SARS-CoV-2 Spike Receptor-Binding Domain Bound to the ACE2 Receptor. Nature 2020, 581, 215–220. 10.1038/s41586-020-2180-5. 32225176

[ref8] Shang J.; Ye G.; Shi K.; Wan Y.; Luo C.; Aihara H.; Geng Q.; Auerbach A.; Li F. Structural Basis of Receptor Recognition by SARS-CoV-2. Nature 2020, 581, 221–224. 10.1038/s41586-020-2179-y. 32225175 PMC7328981

[ref9] Sheahan T. P.; Sims A. C.; Zhou S.; Graham R. L.; Hill C. S.; Leist S. R.; Schäfer A.; Dinnon K. H.; Montgomery S. A.; Agostini M. L.; Pruijssers A. J.; Chapell J. D.; Brown A. J.; Bluemling G. R.; Natchus M. G.; Saindane M.; Kolykhalov A. A.; Painter G.; Harcourt J.; Tamin A.; Thornburg N. J.; Swanstrom R.; Denison M. R.; Baric R. S. An Orally Bioavailable Broad-Spectrum Antiviral Inhibits SARS-CoV-2 and Multiple Endemic, Epidemic and Bat Coronavirus. Sci. Transl. Med 2020, 541, eabb5883. 10.1126/scitranslmed.abb5883 PMC7164393 32253226

[ref10] Vlachakis D.; Papakonstantinou E.; Mitsis T.; Pierouli K.; Diakou I.; Chrousos G.; Bacopoulou F. Molecular Mechanisms of the Novel Coronavirus SARS-CoV-2 and Potential Anti-COVID19 Pharmacological Targets since the Outbreak of the Pandemic. Food Chem. Toxicol. 2020, 146, 111805 10.1016/j.fct.2020.111805. 33038452 PMC7543766

[ref11] Chandra A.; Chaudhary M.; Qamar I.; Singh N.; Nain V. Silico Identification and Validation of Natural Antiviral Compounds as Potential Inhibitors of SARS-CoV-2 Methyltransferase. J. Biomol. Struct. Dyn. 2021, 40, 6534–6544. 10.1080/07391102.2021.1886174. 33583328 PMC7885726

[ref12] Newman J. A.; Douangamath A.; Yadzani S.; Yosaatmadja Y.; Aimon A.; Brandão-Neto J.; Dunnett L.; Gorrie-stone T.; Skyner R.; Fearon D.; Schapira M.; von Delft F.; Gileadi O. Structure, Mechanism and Crystallographic Fragment Screening of the SARS-CoV-2 NSP13 Helicase. Nat. Commun. 2021, 12, 1–11. 10.1038/s41467-021-25166-6. 34381037 PMC8358061

[ref13] Mickolajczyk K. J.; Shelton P. M. M.; Grasso M.; Cao X.; Warrington S. E.; Aher A.; Liu S.; Kapoor T. M. Force-Dependent Stimulation of RNA Unwinding by SARS-CoV-2 Nsp13 Helicase. Biophys. J. 2021, 120, 1020–1030. 10.1016/j.bpj.2020.11.2276. 33340543 PMC7837305

[ref14] Jang K. J.; Jeong S.; Kang D. Y.; Sp N.; Yang Y. M.; Kim D. E. A High ATP Concentration Enhances the Cooperative Translocation of the SARS Coronavirus Helicase NsP13 in the Unwinding of Duplex RNA. Sci. Rep. 2020, 10, 1–13. 10.1038/s41598-020-61432-1. 32161317 PMC7066239

[ref15] Jia Z.; Yan L.; Ren Z.; Wu L.; Wang J.; Guo J.; Zheng L.; Ming Z.; Zhang L.; Lou Z.; Rao Z. Delicate Structural Coordination of the Severe Acute Respiratory Syndrome Coronavirus Nsp13 upon ATP Hydrolysis. Nucleic Acids Res. 2019, 47, 6538–6550. 10.1093/nar/gkz409. 31131400 PMC6614802

[ref16] Chen J.; Malone B.; Llewellyn E.; Grasso M.; Shelton P. M. M.; Olinares P. D. B.; Maruthi K.; Eng E. T.; Vatandaslar H.; Chait B. T.; Kapoor T. M.; Darst S. A.; Campbell E. A. Structural Basis for Helicase-Polymerase Coupling in the SARS-CoV-2 Replication-Transcription Complex. Cell 2020, 182, 1560–1573. 10.1016/j.cell.2020.07.033. 32783916 PMC7386476

[ref17] Ivanov K. A.; Thiel V.; Dobbe J. C.; van der Meer Y.; Snijder E. J.; Ziebuhr J. Multiple Enzymatic Activities Associated with Severe Acute Respiratory Syndrome Coronavirus Helicase. J. Virol. 2004, 78, 5619–5632. 10.1128/jvi.78.11.5619-5632.2004. 15140959 PMC415832

[ref18] Ugurel O. M.; Mutlu O.; Sariyer E.; Kocer S.; Ugurel E.; Inci T. G.; Ata O.; Turgut-Balik D. Evaluation of the Potency of FDA-Approved Drugs on Wild Type and Mutant SARS-CoV-2 Helicase (Nsp13). Int. J. Biol. Macromol. 2020, 163, 1687–1696. 10.1016/j.ijbiomac.2020.09.138. 32980406 PMC7513821

[ref19] White M. A.; Lin W.; Cheng X. Discovery of COVID-19 Inhibitors Targeting the SARS-CoV-2 Nsp13 Helicase. J. Phys. Chem. Lett. 2020, 11, 9144–9151. 10.1021/acs.jpclett.0c02421. 33052685 PMC7571306

[ref20] Gurung A. B. Silico Structure Modelling of SARS-CoV-2 Nsp13 Helicase and Nsp14 and Repurposing of FDA Approved Antiviral Drugs as Dual Inhibitors. Gene reports 2020, 21, 100860 10.1016/j.genrep.2020.100860. 32875166 PMC7452913

[ref21] Zeng H.; Gao X.; Xu G.; Zhang S.; Cheng L.; Xiao T.; Zu W.; Zhang Z. SARS-CoV-2 Helicase NSP13 Hijacks the Host Protein EWSR1 to Promote Viral Replication by Enhancing RNA Unwinding Activity. Infect. Med. 2022, 1 (1), 7–16. 10.1016/j.imj.2021.12.004. PMC8868009 38074973

[ref22] Freidel M. R.; Armen R. S. Mapping major SARS-CoV-2 drug targets and assessment of druggability using computational fragment screening: Identification of an allosteric small-molecule binding site on the Nsp13 helicase. PLOS ONE 2021, 16, e0246181 10.1371/journal.pone.0246181. 33596235 PMC7888625

[ref23] Mirza M. U.; Froeyen M. Structural Elucidation of SARS-CoV-2 Vital Proteins: Computational Methods Reveal Potential Drug Candidates against Main Protease, Nsp12 Polymerase and Nsp13 Helicase. J. Pharm. Anal. 2020, 10, 320–328. 10.1016/j.jpha.2020.04.008. 32346490 PMC7187848

[ref24] Shu T.; Huang M.; Wu D.; Ren Y.; Zhang X.; Han Y.; Mu J.; Wang R.; Qiu Y.; Zhang D.-Y.; Zhou X. SARS-Coronavirus-2 Nsp13 Possesses NTPase and RNA Helicase Activities That Can Be Inhibited by Bismuth Salts. Virol. Sin. 2020, 35, 321–329. 10.1007/s12250-020-00242-1. 32500504 PMC7271831

[ref25] Yuan S.; Wang R.; Chan J. F.-W.; Zhang A. J.; Cheng T.; Chik K. K.-H.; Ye Z.-W.; Wang S.; Lee A. C.-Y.; Jin L.; Li H.; Jin D.-Y.; Yuen K.-Y.; Sun H. Metallodrug Ranitidine Bismuth Citrate Suppresses SARS-CoV-2 Replication and Relieves Virus-Associated Pneumonia in Syrian Hamsters. Nat. Microbiol. 2020, 5, 1439–1448. 10.1038/s41564-020-00802-x. 33028965

[ref26] Yang N.; Tanner J. A.; Zheng B.-J.; Watt R. M.; He M.-L.; Lu L.-Y.; Jiang J.-Q.; Shum K.-T.; Lin Y.-P.; Wong K.-L.; Lin M. C. M.; Kung H.-F.; Sun H.; Huang J.-D. Bismuth Complexes Inhibit the SARS Coronavirus. Angew. Chem., Int. Ed. Engl. 2007, 46, 6464–6468. 10.1002/anie.200701021. 17645269 PMC7159583

[ref27] Malone B.; Chen J.; Wang Q.; Llewellyn E.; Choi Y. J.; Olinares P. D. B.; Cao X.; Hernandez C.; Eng E. T.; Chait B. T.; Shaw D. E.; Landick R.; Darst S. A.; Campbell E. A. Structural Basis for Backtracking by the SARS-CoV-2 Replication-Transcription Complex. Proc. Natl. Acad. Sci. U.S.A. 2021, 118, 2–9. 10.1073/pnas.2102516118. PMC8126829 33883267

[ref28] Chen J.; Wang Q.; Malone B.; Llewellyn E.; Pechersky Y.; Maruthi K.; Eng E. T.; Perry J. K.; Campbell E. A.; Shaw D. E.; Darst S. A. Ensemble Cryo-Electron Microscopy Reveals Conformational States of the Nsp13 Helicase in the SARS-CoV-2 Helicase Replication-Transcription Complex. bioRxiv 2021, 2021, 11 10.1038/s41594-022-00734-6. PMC8935131 35260847

[ref29] Yan L.; Zhang Y.; Ge J.; Zheng L.; Gao Y.; Wang T.; Jia Z.; Wang H.; Huang Y.; Li M.; Wang Q.; Rao Z.; Lou Z. Architecture of a SARS-CoV-2 Mini Replication and Transcription Complex. Nat. Commun. 2020, 11, 3–8. 10.1038/s41467-020-19770-1. 33208736 PMC7675986

[ref30] Tolbatov I.; Marrone A. Computational Strategies to Model the Interaction and the Reactivity of Biologically-Relevant Transition Metal Complexes. Inorganica Chim. Acta 2022, 530, 120686 10.1016/j.ica.2021.120686.

[ref31] Tolbatov I.; Marrone A.; Paciotti R.; Re N.; Coletti C. Multilayered Modelling of the Metallation of Biological Targets. In International Conference on Computational Science and Its Applications; Springer Berlin Heidelberg, 2021, pp 398–412.

[ref32] Storchi L. ATOMIF.Https://Github.Com/Lstorchi/Atomif. 2021

[ref33] Paciotti R.; Storchi L.; Marrone A. Homodimeric complexes of the 90–231 human prion: a multilayered computational study based on FMO/GRID-DRY approach. J. Mol. Model. 2022, 28, 241 10.1007/s00894-022-05244-2. 35918494 PMC9345805

[ref34] Frisch M. J.; Trucks G. W.; Schlegel H. B.; Scuseria G. E.; Robb M. A.; Cheeseman J. R.; Scalmani G.; Barone V.; Mennucci B.; Petersson G. A.; Nakatsuji H.; Caricato M.; Li X.; Hratchian H. P.; Izmaylov A. F.; Bloino J.; Zheng G.; Sonnenberg J. L.; Had M.; Gaussian 09; Gaussian, Inc.: Wallingford CT, 2009.

[ref35] Chai J.-D.; Head-Gordon M. Systematic Optimization of Long-Range Corrected Hybrid Density Functionals. J. Chem. Phys. 2008, 128, 084106 10.1063/1.2834918. 18315032

[ref36] Weigend F.; Ahlrichs R. Balanced Basis Sets of Split Valence, Triple Zeta Valence and Quadruple Zeta Valence Quality for H to Rn: Design and Assessment of Accuracy. Phys. Chem. Chem. Phys. 2005, 7, 3297–3305. 10.1039/b508541a. 16240044

[ref37] Todisco S.; Latronico M.; Gallo V.; Re N.; Marrone A.; Tolbatov I.; Mastrorilli P. Double Addition of Phenylacetylene onto the Mixed Bridge Phosphinito-Phosphanido Pt(i) Complex [(PHCy2)Pt(μ-PCy2){κ2: P, O -μ-P(O)Cy2}Pt(PHCy2)](Pt-Pt). Dalt. Trans. 2020, 49, 6776–6789. 10.1039/d0dt00923g. 32374320

[ref38] Paciotti R.; Tolbatov I.; Marrone A.; Storchi L.; Re N.; Coletti C. Computational Investigations of Bioinorganic Complexes: The Case of Calcium, Gold and Platinum Ions. AIP Conf. Proc. 2019, 2186, 30011 10.1063/1.5137922.

[ref39] Marrone A.; Fish R. H. DFT Mechanism Studies: Biomimetic 1,4-NADH Chemoselective, Co-Factor Regeneration with [Cp*Rh(Bpy)H]+, in Tandem with the Biocatalysis Pathways of a Core Model of the (HLADH)-Zn(II) Mediated Enzyme, in the Enantioselective Reduction of Achiral Ketones To. J. Organomet. Chem. 2021, 943, 121810 10.1016/j.jorganchem.2021.121810.

[ref40] Tolbatov I.; Marrone A. Molecular Dynamics Simulation of the Pb(II) Coordination in Biological Media via Cationic Dummy Atom Models. Theor. Chem. Acc. 2021, 140, 1–12. 10.1007/s00214-021-02718-z.

[ref41] Tolbatov I.; Coletti C.; Marrone A.; Re N. Reactivity of Arsenoplatin Complex versus Water and Thiocyanate: A DFT Benchmark Study. Theor. Chem. Acc. 2020, 139, 1–11. 10.1007/s00214-020-02694-w.

[ref42] Tolbatov I.; Marzo T.; Coletti C.; La Mendola D.; Storchi L.; Re N.; Marrone A. Reactivity of Antitumor Coinage Metal-Based N-Heterocyclic Carbene Complexes with Cysteine and Selenocysteine Protein Sites. J. Inorg. Biochem. 2021, 223, 111533 10.1016/j.jinorgbio.2021.111533. 34273714

[ref43] Barresi E.; Tolbatov I.; Marzo T.; Zappelli E.; Marrone A.; Re N.; Pratesi A.; Martini C.; Taliani S.; Da Settimo F.; La Mendola D. Two Mixed Valence Diruthenium(Ii,Iii) Isomeric Complexes Show Different Anticancer Properties. Dalt. Trans. 2021, 50, 9643–9647. 10.1039/d1dt01492g. 34160519

[ref44] Dohm S.; Hansen A.; Steinmetz M.; Grimme S.; Checinski M. P. Comprehensive Thermochemical Benchmark Set of Realistic Closed-Shell Metal Organic Reactions. J. Chem. Theory Comput. 2018, 14, 2596–2608. 10.1021/acs.jctc.7b01183. 29565586

[ref45] Sullivan M. P.; Cziferszky M.; Tolbatov I.; Truong D.; Mercadante D.; Re N.; Gust R.; Goldstone D. C.; Hartinger C. G. Probing the Paradigm of Promiscuity for N-Heterocyclic Carbene Complexes and Their Protein Adduct Formation. Angew. Chemie - Int. Ed. 2021, 60, 19928–19932. 10.1002/anie.202106906. 34196088

[ref46] Cossi M.; Rega N.; Scalmani G.; Barone V. Energies, Structures, and Electronic Properties of Molecules in Solution with the C-PCM Solvation Model. J. Comput. Chem. 2003, 24, 669–681. 10.1002/jcc.10189. 12666158

[ref47] Storchi L. ATOMIF.

[ref48] Duan Y.; Wu C.; Chowdhury S.; Lee M. C.; Xiong G.; Zhang W.; Yang R.; Cieplak P.; Luo R.; Lee T.; Caldwell J.; Wang J.; Kollman P. A Point-Charge Force Field for Molecular Mechanics Simulations of Proteins Based on Condensed-Phase Quantum Mechanical Calculations. J. Comput. Chem. 2003, 24, 1999–2012. 10.1002/jcc.10349. 14531054

[ref49] Van Der Spoel D.; Lindahl E.; Hess B.; Groenhof G.; Mark A. E.; Berendsen H. J. C. GROMACS: Fast, Flexible, and Free. J. Comput. Chem. 2005, 26, 1701–1718. 10.1002/jcc.20291. 16211538

[ref50] Berendsen H. J. C.; Postma J. P. M.; van Gunsteren W. F.; DiNola A.; Haak J. R. Molecular Dynamics with Coupling to an External Bath. J. Chem. Phys. 1984, 81, 3684–3690. 10.1063/1.448118.

[ref51] Hess B.; P-LINCS B. A Parallel Linear Constraint Solver for Molecular Simulation. J. Chem. Theory Comput. 2008, 4, 116–122. 10.1021/ct700200b. 26619985

[ref52] Darden T.; York D.; Pedersen L. Particle Mesh Ewald: An N·log(N) Method for Ewald Sums in Large Systems. J. Chem. Phys. 1993, 98, 10089–10092. 10.1063/1.464397.

[ref53] Humphrey W.; Dalke A.; Schulten K. Sartorius Products. J. Mol. Graph. 1996, 14, 33–38. 10.1016/0263-7855(96)00018-5. 8744570

[ref54] Schrödinger L. L. C. Maestro, Version 9.4: New York, NY, U. No Title, 2013.

[ref55] Daura X.; Gademann K.; Jaun B.; Seebach D.; Van Gunsteren W. F.; Mark A. E. Peptide Folding: When Simulation Meets Experiment. Angew. Chemie - Int. Ed. 1999, 38, 236–240. 10.1002/(sici)1521-3773(19990115)38:1/2<236::aid-anie236>3.0.co;2-m.

[ref56] Tortorella S.; Carosati E.; Sorbi G.; Bocci G.; Cross S.; Cruciani G.; Storchi L. Combining Machine Learning and Quantum Mechanics Yields More Chemically Aware Molecular Descriptors for Medicinal Chemistry Applications. J. Comput. Chem. n 2021, 42, 26737 10.1002/jcc.26737. PMC9291213 34410004

[ref57] Jurrus E.; Engel D.; Star K.; Monson K.; Brandi J.; Felberg L. E.; Brookes D. H.; Wilson L.; Chen J.; Liles K.; Chun M.; Li P.; Gohara D. W.; Dolinsky T.; Konecny R.; Koes D. R.; Nielsen J. E.; Head-Gordon T.; Geng W.; Krasny R.; Wei G.-W.; Holst M. J.; McCammon J. A.; Baker N. A. Improvements to the APBS Biomolecular Solvation Software Suite. Protein Sci. 2018, 27, 112–128. 10.1002/pro.3280. 28836357 PMC5734301

[ref58] Konecny R.; Baker N. A.; McCammon J. A. IAPBS: A Programming Interface to the Adaptive Poisson-Boltzmann Solver. Comput. Sci. Discov. 2012, 5, 015005 10.1088/1749-4699/5/1/015005. 22905037 PMC3419494

[ref59] Agamennone M.; Storchi L.; Marrone A.; Paciotti R. Hampering the Early Aggregation of PrP - E200K Protein by Charge - Based Inhibitors : A Computational Study. J. Comput. Aided. Mol. Des. 2021, 35, 393 10.1007/s10822-021-00393-7. PMC8213589 34110550

[ref60] Paciotti R.; Storchi L.; Marrone A. An Insight of Early PrP-E200K Aggregation by Combined Molecular Dynamics/Fragment Molecular Orbital Approaches. Proteins Struct. Funct. Bioinforma. 2019, 87, 51–61. 10.1002/prot.25621. 30367504

[ref61] Storchi L.; Paciotti R.; Re N.; Marrone A. Investigation of the Molecular Similarity in Closely Related Protein Systems: The PrP Case Study. Proteins Struct. Funct. Bioinforma. 2015, 83, 1751–1765. 10.1002/prot.24836. 26018750

[ref62] Cirri D.; Marzo T.; Tolbatov I.; Marrone A.; Saladini F.; Vicenti I.; Dragoni F.; Boccuto A.; Messori L. Vitro Anti-Sars-Cov-2 Activity of Selected Metal Compounds and Potential Molecular Basis for Their Actions Based on Computational Study. Biomolecules 2021, 11, 11121858 10.3390/biom11121858. PMC8699537 34944502

[ref63] de Paiva R. E. F.; Marçal Neto A.; Santos I. A.; Jardim A. C. G.; Corbi P. P.; Bergamini F. R. G. What Is Holding Back the Development of Antiviral Metallodrugs? A Literature Overview and Implications for SARS-CoV-2 Therapeutics and Future Viral Outbreaks. Dalt. Trans. 2020, 49, 16004–16033. 10.1039/d0dt02478c. 33030464

[ref64] Udalova T. A.; Logutenko O. A.; Timakova E. V.; Afonina L. I.; Naydenko E. S.; Yukhin Y. M.. Bismuth Compounds in Medicine; International Forum on Strategic Technologies, 2008, pp 137–140.

[ref65] Yang N.; Sun H. Biocoordination Chemistry of Bismuth: Recent Advances. Coord. Chem. Rev. 2007, 251, 2354–2366. 10.1016/j.ccr.2007.03.003.

