# Supplementary material for: Structural Reshaping of the Zinc-Finger Domain of the SARS-CoV-2 nsp13 Protein Using Bismuth(III) Ions: A Multilevel Computational Study
Source: Inorg Chem. 2022 Sep 20;61(39):15664–77. doi: 10.1021/acs.inorgchem.2c02685 (PMC9514052; doi:10.1021/acs.inorgchem.2c02685)
Supplement: Supplementary file 1 — ic2c02685_si_001.pdf [file ic2c02685_si_001.pdf]

SUPPORTING INFORMATION

Structural reshaping of the zinc-finger domain of the SARS-CoV-2 nsp13 protein by bismuth(III) ions: A multilevel computational study

*Iogann Tolbatov [1], Loriano Storchi [2], Alessandro Marrone [2]**

[1] Institut de Chimie Moleculaire de l'Université de Bourgogne (ICMUB), Université de Bourgogne Franche-Comté (UBFC), avenue Alain Savary 9, Dijon, France

[2] Dipartimento di Farmacia, Università "G d'Annunzio" di Chieti-Pescara, via dei Vestini 31, Chieti, Italy.

** Please correspond to: amarrone@unich.it*

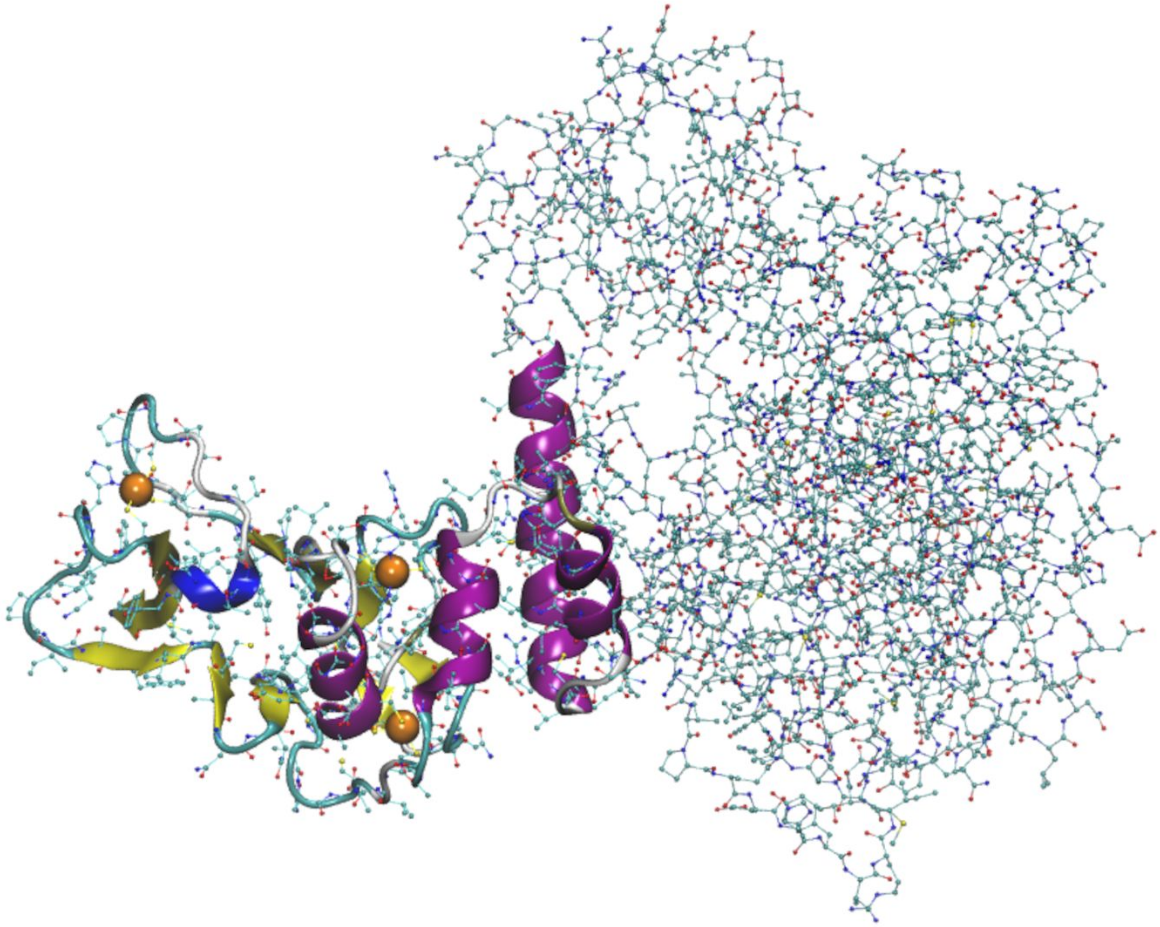

Figure S1. Rendition of the xray structure of nsp13 (entry pdb 6zsl, chain B). The zinc binding domain (ZBD) are displayed with cartoon models. The Zn2+ ions are also represented (orange spheres).

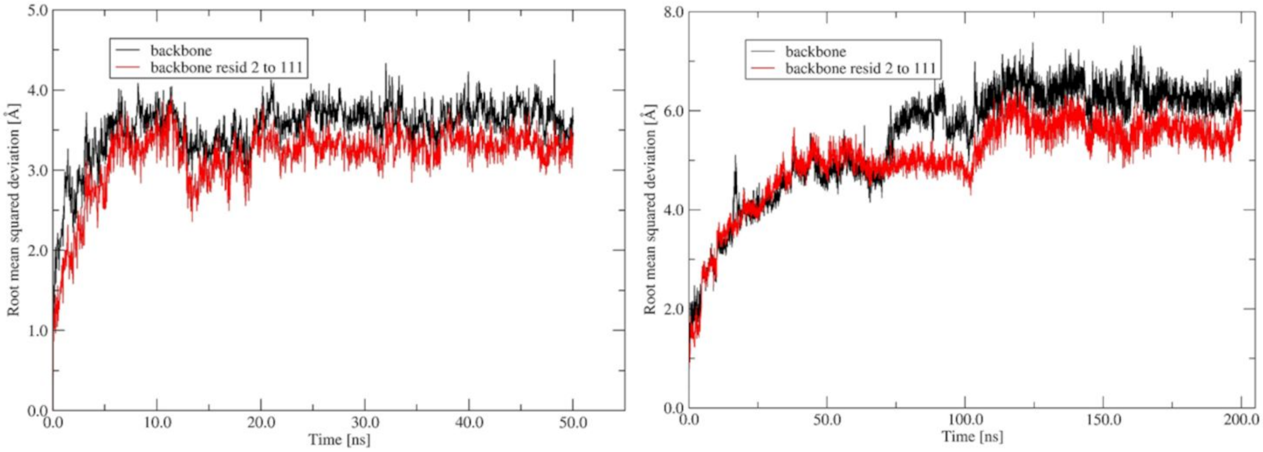

Figure S2. Root mean square deviations for the whole (black) and the segment 2-111 (red) backbone atoms calculated from the Zn-bound (left) and Bi-bound (right) trajectories.

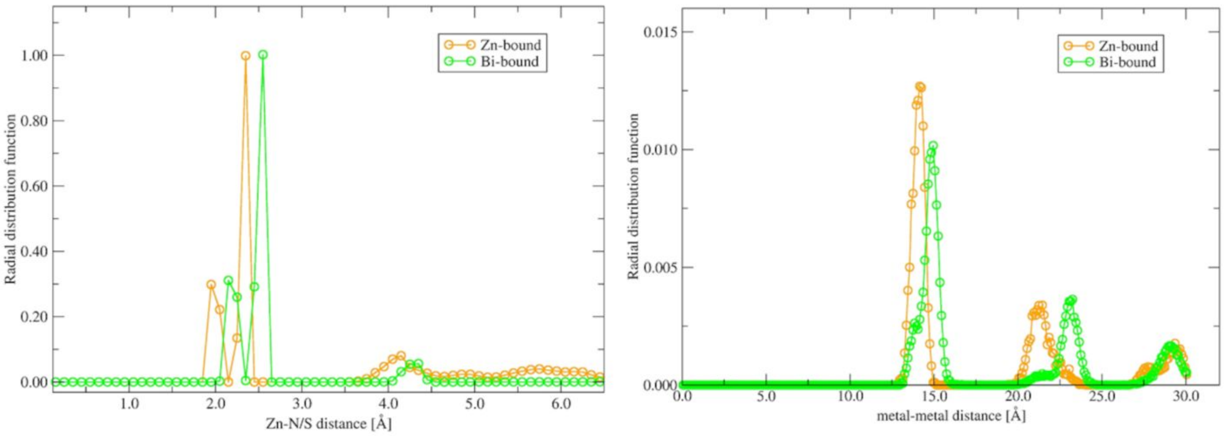

Figure S3. Radial distribution function (rdf) analyses of the Zn-bound (orange) and Bi-bound (green) trajectories. Left: rdf analysis of the first coordination sphere, i.e. analysis of metal-S_{CYS} or metal-N_{HIS} distances; rdf values were rescaled over the global maximum value (assigned rdf=1.0). Right: rdf analysis of the metal-metal distance.

Table S1. Decomplexation, metal exchange, and residue interaction energies (both metal-bound and apo models) of the ZF1-3 domain of nsp13 calculated at DFT level of theory.

Domain	Metal coordination	decomplexation		exchange	Residue interaction energy	
		Zn-bound	Bi-bound	$Zn^{2+} \rightarrow Bi^{3+}$	Zn (apo)	Bi (apo)
ZF1	$(SH)_4$	94.7	242.1	-145.9	-89.2 (5.5)	-235.1 (7.0)
	$(S^-)(SH)_3$	180.8	343.3	-155.0	-180.3 (0.4)	-335.3 (8.0)
	$(S^-)_2(SH)_2$	249.5	470.2	-217.1	-228.7 (20.8)	-445.8 (24.4)
	$(S^-)_3(SH)$	306.0	564.1	-244.5	-260.0 (46.0)	-504.6 (59.6)
	$(S^-.)_4$	365.3	633.3	-275.3	-244.9 (120.4)	-520.2 (113.1)
ZF2	$N_2(SH)_2$	153.8	305.0	-144.8	-146.2 (7.6)	-291.0 (14.0)
	$N_2(S^-)(SH)$	223.6	420.9	-191.6	-210.2 (13.4)	-401.8 (19.1)
	$N_2(S^-)_2$	289.4	512.7	-219.0	-249.7 (39.7)	-468.7 (44.1)
ZF3	$N(SH)_3$	125.5	274.1	-157.9	-108.6 (16.9)	-266.5 (7.6)
	$N(S^-)(SH)_2$	200.8	383.3	-176.4	-191.9 (8.9)	-368.3 (15.0)
	$N(S^-)_2(SH)$	269.2	499.8	-223.7	-237.4 (31.8)	-461.2 (38.6)
	$N(S^-)_3$	330.8	579.3	-248.9	-258.8 (72.0)	-507.6 (71.7)

Table S2. Stride analysis of the secondary structures of nsp13 ZBD binding either Zn^{2+} or Bi^{3+} ions. Helices and β-sheet domains are reported in bold. The loss of secondary structure (either helix or β-sheet) compared to the xray model was 17.6% and 27.9% for the most representative conformations of Zn-bound and Bi-bound models, respectively.

residue	chain	resid	Xray	Zn-bound	Bi-bound
VAL	B	2	Coil	Coil	Coil
GLY	B	3	**Strand**	**Strand**	Coil
ALA	B	4	**Strand**	**Strand**	Coil
CYS	B	5	Turn	**Strand**	Bridge
VAL	B	6	Turn	Turn	Turn
LEU	B	7	Turn	Turn	Turn
CYS	B	8	Turn	Turn	Turn
ASN	B	9	Turn	Turn	Coil
SER	B	10	Coil	**Strand**	Coil
GLN	B	11	**Strand**	**Strand**	Coil
THR	B	12	**Strand**	**Strand**	Coil
SER	B	13	Coil	Coil	Coil
LEU	B	14	Coil	Coil	**Strand**
ARG	B	15	**Strand**	**Strand**	**Strand**
CYS	B	16	**Strand**	**Strand**	Turn
GLY	B	17	Turn	Turn	Turn
ALA	B	18	Turn	Turn	Turn

CYS	B	19	Turn	Turn	Turn
ILE	B	20	Turn	Turn	Coil
ARG	B	21	Turn	Turn	Coil
ARG	B	22	Turn	Turn	Coil
PRO	B	23	Coil	Coil	Coil
PHE	B	24	Bridge	Bridge	Coil
LEU	B	25	Coil	Coil	Coil
CYS	B	26	Coil	Coil	Coil
CYS	B	27	**AlphaHelix**	**AlphaHelix**	**AlphaHelix**
LYS	B	28	**AlphaHelix**	**AlphaHelix**	**AlphaHelix**
CYS	B	29	**AlphaHelix**	**AlphaHelix**	**AlphaHelix**
CYS	B	30	**AlphaHelix**	**AlphaHelix**	**AlphaHelix**
TYR	B	31	**AlphaHelix**	**AlphaHelix**	**AlphaHelix**
ASP	B	32	**AlphaHelix**	**AlphaHelix**	**AlphaHelix**
HIS	B	33	**AlphaHelix**	**AlphaHelix**	**AlphaHelix**
VAL	B	34	**AlphaHelix**	**AlphaHelix**	**AlphaHelix**
ILE	B	35	**AlphaHelix**	**AlphaHelix**	**AlphaHelix**
SER	B	36	Coil	**AlphaHelix**	**AlphaHelix**
THR	B	37	Coil	Coil	Coil
SER	B	38	Coil	Coil	Coil
HIS	B	39	Coil	Coil	Coil
LYS	B	40	Coil	Coil	Coil
LEU	B	41	Coil	Coil	Coil
VAL	B	42	**Strand**	**Strand**	Coil
LEU	B	43	**Strand**	**Strand**	**Strand**
SER	B	44	Turn	Turn	**Strand**
VAL	B	45	Turn	Turn	Coil
ASN	B	46	Turn	Turn	Coil
PRO	B	47	**Strand**	Turn	Coil
TYR	B	48	**Strand**	Coil	Bridge
VAL	B	49	Turn	Coil	Coil
CYS	B	50	Coil	Coil	Turn
ASN	B	51	Coil	Coil	Turn
ALA	B	52	Turn	Turn	Turn
PRO	B	53	Turn	Turn	Turn
GLY	B	54	Turn	Turn	Coil
CYS	B	55	Turn	Turn	Coil
ASP	B	56	Coil	Coil	Coil
VAL	B	57	Coil	Coil	Coil
THR	B	58	Coil	Coil	Coil
ASP	B	59	Coil	Turn	Turn
VAL	B	60	310Helix	Turn	Turn
THR	B	61	310Helix	Turn	Turn

GLN	B	62	310Helix	Turn	Turn
LEU	B	63	Coil	**Strand**	**Strand**
TYR	B	64	**Strand**	**Strand**	**Strand**
LEU	B	65	**Strand**	**Strand**	**Strand**
GLY	B	66	**Strand**	Turn	Turn
GLY	B	67	Turn	Turn	Turn
MET	B	68	Turn	Turn	Turn
SER	B	69	**Strand**	Turn	Turn
TYR	B	70	**Strand**	**Strand**	**Strand**
TYR	B	71	**Strand**	**Strand**	**Strand**
CYS	B	72	Turn	**Strand**	**Strand**
LYS	B	73	Turn	Turn	Turn
SER	B	74	Turn	Turn	Turn
HIS	B	75	Turn	Turn	Turn
LYS	B	76	Turn	Turn	Turn
PRO	B	77	Turn	Turn	Turn
PRO	B	78	Turn	Turn	Turn
ILE	B	79	Turn	Turn	Turn
SER	B	80	**Strand**	Turn	Turn
PHE	B	81	**Strand**	**Strand**	**Strand**
PRO	B	82	**Strand**	**Strand**	**Strand**
LEU	B	83	Turn	Coil	Turn
CYS	B	84	**Strand**	**Strand**	**Strand**
ALA	B	85	**Strand**	**Strand**	**Strand**
ASN	B	86	Turn	Turn	Turn
GLY	B	87	Turn	Turn	Turn
GLN	B	88	**Strand**	**Strand**	**Strand**
VAL	B	89	**Strand**	**Strand**	**Strand**
PHE	B	90	Coil	Coil	Coil
GLY	B	91	Turn	Coil	Coil
LEU	B	92	Turn	Coil	Coil
TYR	B	93	Turn	Coil	Coil
LYS	B	94	Turn	Coil	**AlphaHelix**
ASN	B	95	Turn	Coil	**AlphaHelix**
THR	B	96	Turn	Coil	**AlphaHelix**
CYS	B	97	Turn	Coil	**AlphaHelix**
VAL	B	98	Coil	Coil	Turn
GLY	B	99	Coil	Coil	Turn
SER	B	100	Turn	Coil	Turn
ASP	B	101	Turn	Coil	Turn
ASN	B	102	Turn	Turn	Turn
VAL	B	103	**AlphaHelix**	Turn	Bridge
THR	B	104	**AlphaHelix**	Turn	Turn

ASP	B	105	**AlphaHelix**	Turn	Turn
PHE	B	106	**AlphaHelix**	**AlphaHelix**	**Turn**
ASN	B	107	**AlphaHelix**	**AlphaHelix**	**Turn**
ALA	B	108	**AlphaHelix**	**AlphaHelix**	**Turn**
ILE	B	109	**AlphaHelix**	**AlphaHelix**	**Turn**
ALA	B	110	**AlphaHelix**	**AlphaHelix**	**Turn**
THR	B	111	**AlphaHelix**	**AlphaHelix**	**Turn**
CYS	B	112	Coil	Coil	Turn
ASP	B	113	Coil	Coil	Turn
TRP	B	114	Coil	Turn	Turn
THR	B	115	Coil	Turn	Coil
ASN	B	116	Bridge	Turn	Coil
ALA	B	117	**AlphaHelix**	**AlphaHelix**	**AlphaHelix**
GLY	B	118	**AlphaHelix**	**AlphaHelix**	**AlphaHelix**
ASP	B	119	**AlphaHelix**	**AlphaHelix**	**AlphaHelix**
TYR	B	120	**AlphaHelix**	**AlphaHelix**	**AlphaHelix**
ILE	B	121	**AlphaHelix**	**AlphaHelix**	**AlphaHelix**
LEU	B	122	**AlphaHelix**	**AlphaHelix**	Turn
ALA	B	123	**AlphaHelix**	**AlphaHelix**	Turn
ASN	B	124	**AlphaHelix**	**AlphaHelix**	Turn
THR	B	125	Coil	**AlphaHelix**	Turn
CYS	B	126	Coil	Coil	Turn
THR	B	127	Coil	Turn	Turn
GLU	B	128	**AlphaHelix**	Turn	Turn
ARG	B	129	**AlphaHelix**	Turn	Turn
LEU	B	130	**AlphaHelix**	**AlphaHelix**	**AlphaHelix**
LYS	B	131	**AlphaHelix**	**AlphaHelix**	**AlphaHelix**
LEU	B	132	**AlphaHelix**	**AlphaHelix**	**AlphaHelix**
PHE	B	133	**AlphaHelix**	**AlphaHelix**	**AlphaHelix**
ALA	B	134	**AlphaHelix**	**AlphaHelix**	**AlphaHelix**
ALA	B	135	**AlphaHelix**	**AlphaHelix**	**AlphaHelix**
GLU	B	136	**AlphaHelix**	**AlphaHelix**	**AlphaHelix**
THR	B	137	**AlphaHelix**	**AlphaHelix**	**AlphaHelix**
LEU	B	138	**AlphaHelix**	**AlphaHelix**	**AlphaHelix**
LYS	B	139	**AlphaHelix**	Turn	**AlphaHelix**
ALA	B	140	**AlphaHelix**	Turn	**AlphaHelix**
THR	B	141	**AlphaHelix**	Turn	**AlphaHelix**
GLU	B	142	**AlphaHelix**	Turn	**AlphaHelix**
GLU	B	143	**AlphaHelix**	Turn	**AlphaHelix**
THR	B	144	**AlphaHelix**	Turn	Coil
PHE	B	145	**AlphaHelix**	Coil	Coil
LYS	B	146	**AlphaHelix**	Coi	Coi
